# *Leishmania* exploits host cAMP/EPAC/calcineurin signaling to induce an IL-33–mediated anti-inflammatory environment for the establishment of infection

**DOI:** 10.1016/j.jbc.2024.107366

**Published:** 2024-05-13

**Authors:** Souravi Roy, Shalini Roy, Satyajit Halder, Kuladip Jana, Anindita Ukil

**Affiliations:** 1 Department of Biochemistry, University of Calcutta, Kolkata, India; 2 Division of Molecular Medicine, Bose Institute, Kolkata, India

**Keywords:** macrophage, *Leishmania*, IL-33, signaling, cAMP, EPAC, calcineurin, transcription factor

## Abstract

Host anti-inflammatory responses are critical for the progression of visceral leishmaniasis, and the pleiotropic cytokine interleukin (IL)-33 was found to be upregulated in infection. Here, we documented that IL-33 induction is a consequence of elevated cAMP-mediated exchange protein activated by cAMP (EPAC)/calcineurin-dependent signaling and essential for the sustenance of infection*. Leishmania donovani–*infected macrophages showed upregulation of IL-33 and its neutralization resulted in decreased parasite survival and increased inflammatory responses. Infection-induced cAMP was involved in IL-33 production and of its downstream effectors PKA and EPAC, only the latter was responsible for elevated IL-33 level. EPAC initiated Rap-dependent phospholipase C activation, which triggered the release of intracellular calcium followed by calcium/calmodulin complex formation. Screening of calmodulin-dependent enzymes affirmed involvement of the phosphatase calcineurin in cAMP/EPAC/calcium/calmodulin signaling–induced IL-33 production and parasite survival. Activated calcineurin ensured nuclear localization of the transcription factors, nuclear factor of activated T cell 1 and hypoxia-inducible factor 1 alpha required for IL-33 transcription, and we further confirmed this by chromatin immunoprecipitation assay. Administering specific inhibitors of nuclear factor of activated T cell 1 and hypoxia-inducible factor 1 alpha in BALB/c mouse model of visceral leishmaniasis decreased liver and spleen parasite burden along with reduction in IL-33 level. Splenocyte supernatants of inhibitor-treated infected mice further documented an increase in tumor necrosis factor alpha and IL-12 level with simultaneous decrease of IL-10, thereby indicating an overall disease-escalating effect of IL-33. Thus, this study demonstrates that cAMP/EPAC/calcineurin signaling is crucial for the activation of IL-33 and in effect creates anti-inflammatory responses, essential for infection.

Interleukin-33 (IL-33) is a recently discovered member of the IL-1 cytokine family (1), which is mainly composed of two domains, of which the N-terminal domain is the nuclear domain and the C-terminal domain is the IL-1–like cytokine domain. It can act both as a chromatin-binding factor and as a cytokine (2). During its nuclear localization, IL-33 functions as a transcriptional regulator of various genes such as NF-κB, intercellular adhesion molecule-1, vascular cell adhesion molecule 1, *etc.* (3). IL-33 acts through a heterodimer composed of the ST2-specific receptor (IL-1RL1) and the IL-1 receptor accessory protein (4, 5). It also functions as an “alarmin,” which is released in response to cell necrosis for alerting the immune system towards tissue damage or stress (6, 7). It is mainly produced by endothelial cells, epithelial cells, fibroblast-like cells, and myofibroblasts, though macrophages and mast cells also produce this cytokine (2). Being pleiotropic, IL-33 participates in the activation of T helper type 2 (Th2) cells, Th1 cells, group 2 innate lymphoid cells, and regulatory T cells (8, 9). In atherosclerosis, IL-33 treatment resulted in the augmentation of Th2 cytokines and reduced the level of the Th1 cytokine interferon-gamma (10). IL-33 also favors ulcerative colitis as shown in the experimental model by activating Th2 responses (11). On the contrary, some studies reported the role of IL-33 in inducing Th1 immune responses as well as Th1 cell differentiation (11, 12). It is, therefore, obvious that IL-33 can activate a variety of signaling cascades depending on its multidimensional ability to develop immune cell activation.

Visceral leishmaniasis (VL), caused by the protozoan parasite *Leishmania donovani* is a fatal disease that affects individuals with poor nutritional status (13) and is a threat to many third-world countries, specifically Brazil, Sudan, and the Indian subcontinent (14). Timely intervention is essential to control the disease and increasing drug resistance against currently available medications further complicates the situation (15). In VL, the cytokine environment within the host largely determines the disease outcome (16) and understanding the pathways that regulate cytokines will shed light on developing potential therapeutics. However, several studies are being carried out to understand the specific roles of various cytokines in immunomodulation during *L. donovani* infection, but to date only a few reports are available as far as IL-33 is concerned (17, 18). Serum IL-33 level was found to be higher in patients with VL as well as in *Leishmania donvani–*infected BALB/c mice than normal uninfected controls. Further, the observation defining better control of hepatic parasitic burden by impairment of IL-33/ST2 signaling points toward a favorable role of IL-33. The absence of ST2 also led to the upregulation of several Th1 cytokines like IL-12 and interferon-gamma suggesting thereby the negative regulation of these cytokines by IL-33 (17). A few other infections like *Plasmodium* sp, *Mycobacterium* sp.*,* and *Trypanosoma* sp. were also found to be associated with the induction of IL-33 (19, 20, 21). However, no study in detail has yet been carried out on how IL-33 itself is induced in macrophages.

The “second messenger” cAMP has been reported to suppress the microbicidal capacity of leukocytes toward various pathogens including bacteria (22, 23, 24), viruses (25), fungi (26), and eukaryotic parasites (27) though the mechanisms are not well elucidated. Signal transmission by cAMP occurs through the activation of downstream effector proteins, cyclic nucleotide-gated ion channels, exchange protein activated by cAMP (EPAC), and PKA. Our previous work documented that in VL, cAMP, and its effector molecules, PKA, and EPAC play a crucial role in the establishment of infection (28). We could identify only the chemokine-reducing activity of EPAC in infected cells and did not look into its contribution towards modulating IL-33. A very recent report suggested that prostaglandin E2-derived cAMP modulated IL-33 in macrophages (29) and another report showed that IL-33 is a critical component for the survival of *Leishmania* (17)*.* We, therefore, thought it worthwhile to look into the role of cAMP signaling in IL-33 induction in VL.

In the present study, we tried to delineate the detailed signaling cascade induced by *Leishmania* to activate IL-33 production. We found that the cAMP/EPAC1/phospholipase C (PLC)/calcium axis primarily regulates IL-33 induction in infected macrophages. *In vitro* and *in vivo* experiments further identified the transcription factors nuclear factor of activated T cell 1 (NFATc1) and hypoxia-inducible factor 1 alpha (HIF-1α), which are activated by calcium-dependent phosphatase calcineurin and lead to IL-33 production during infection. IL-33 shifts the host’s immune balance toward Th2 cytokine profile and the overall mechanism may be utilized by other intramacrophage pathogens also.

## Results

### *L. donovani* infection induces IL-33 production in macrophages to facilitate parasite survival

**Figure 1:**
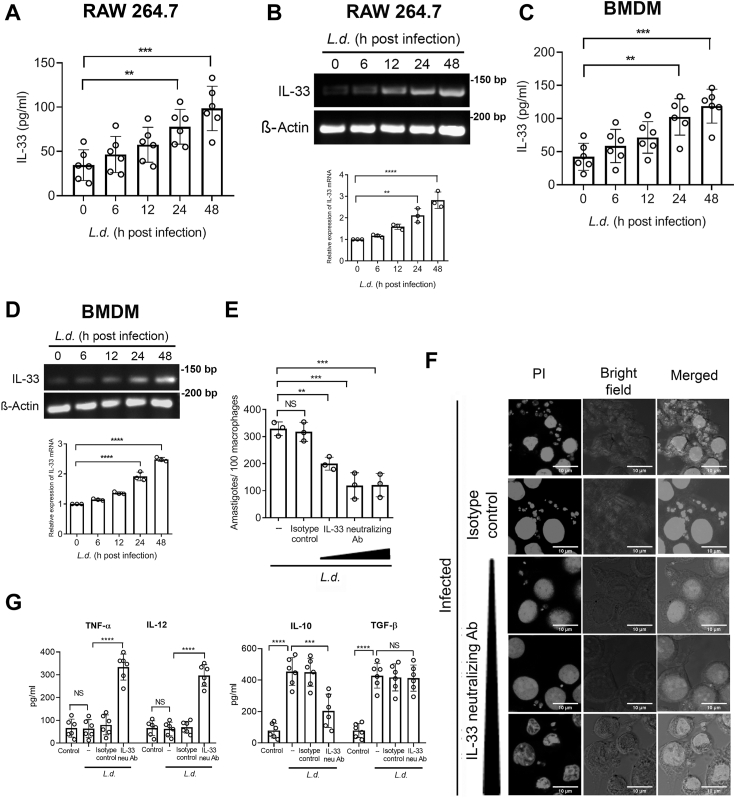
***Leishmania donovani* infection induces IL-33 production in macrophages to facilitate parasite survival.***A* and *B*, RAW 264.7 macrophages were infected with *L. donovani* promastigotes for the indicated time points (0–48 h), and expression of IL-33 was evaluated at the protein level by ELISA (n = 6) and at mRNA level by RT-PCR (n = 3). Statistical significance has been marked relative to uninfected control. *C* and *D*, IL-33 level was also measured at protein (n = 6) and mRNA levels (n = 3) in infected BMDM. Statistical significance has been marked relative to uninfected control. *E* and *F*, RAW 264.7 cells were infected with *L. donovani* promastigotes either in the presence or absence of an IL-33 neutralizing antibody (48 h). Immunoglobulin G was used as an isotype control. The number of parasites per 100 macrophages was determined by PI staining (n = 3), and representative confocal microscopic images are depicted. *G*, culture supernatants of macrophages infected with *L. donovani* promastigotes in the presence or absence of IL-33 neutralizing antibody for 48 h were assayed for TNF-α, IL-12, IL-10, and TGF-β by ELISA (n = 6). The *graph* shows the combined (mean) outcomes from indicated number of independent experiments, and the error bars indicate the variation between those independent repeats (mean ± SD); NS, not significant, ∗*p* < 0.05, ∗∗*p* < 0.01, ∗∗∗*p* < 0.001, and ∗∗∗∗*p* < 0.0001 (ANOVA with Tukey post hoc test). BMDM, bone marrow–derived macrophage; IL, interleukin; PI, propidium iodide; TGF-β, transforming growth factor-beta.

For their successful intramacrophage survival, *Leishmania* facilitates M2 polarization of host macrophage (30), thereby creating an anti-inflammatory environment (31). Since secretion of IL-33 from M2 macrophages has recently been reported to be capable of inducing Th2 immune response (32), therefore, we wanted to determine whether *L. donovani* induces IL-33 production. RAW 264.7 macrophages were subjected to parasite infection for various periods and IL-33 level was determined by ELISA. RAW 264.7 cells were chosen as the cells show similarity to bone marrow–derived macrophages (BMDMs) in terms of surface receptors and response to microbial ligands that induce cellular activation *via* toll-like receptors (TLRs) 3 and 4 (33). A time-dependent increase of IL-33 was observed with a maximum level of 2.9-fold increase compared with uninfected cells at 48 h postinfection (*p* = 0.000102, F _(4, 25)_ = 9.21) (Fig. 1*A*), which started to decrease after 72 h (data not shown). Next, to determine whether this upregulation is reflected in the transcriptional level, mRNA expression of IL-33 in infected macrophages was determined by RT-PCR. Similar to the protein level, maximum expression of IL-33 mRNA was also observed at 48 h postinfection (a 2.8-fold increase compared with uninfected cells, *p* < 0.0001, F _(4, 10)_ = 30.79) (Fig. 1*B*). These observations were further validated in BMDM where a similar trend was obtained in both protein and mRNA levels (Fig. 1, *C* and *D*). To ascertain whether infection-induced IL-33 has any effect on intramacrophage survival of the parasite, infected cells were incubated with graded concentration (5 ng/ml–15 ng/ml) of IL-33 neutralizing antibody and intracellular parasite number was determined by propidium iodide (PI) staining. immunoglobulin G (IgG) was administered (15 ng/ml) as an isotype control. Neutralizing antibodies did not have any apparent effect on the viability of the cells (Fig. S1*A*). Treatment with anti-IL-33 antibody (10 ng/ml) led to a significant reduction in parasite count compared to infected control (63.3% decrease at 48 h postinfection, *p* < 0.0001, F _(4, 10)_ = 23.79) (Fig. 1, *E* and *F*), indicating its importance on parasite survival. Cells pretreated with IL-33 antibody did not document any significant change in the rate of infection as well (Fig. S1*B*). As the cytokine profile of the host cells is one of the most vital parameters that determine the disease outcome (16), therefore we examined whether IL-33 can influence the production of other proinflammatory and anti-inflammatory cytokines that may aid in parasite survival. Inhibition of IL-33 by using neutralizing antibody (10 ng/ml) significantly upregulated the inflammatory cytokines TNF-α (5.2-fold, *p* < 0.0001, F _(3, 20)_ = 50.26) and IL-12 (4.8-fold, *p* < 0.0001, F _(3, 20)_ = 65.52) compared with the very low level found in infected macrophages. On the other hand, neutralizing antibodies significantly downregulated infection-induced IL-10 (54.9% reduction compared to infected cells, *p* < 0.0001, F _(3, 20)_ = 28.47) (Fig. 1*G*). IgG was administered at a concentration of 10 ng/ml as an isotype control. Activation or deactivation of these cytokines was shown to be well correlated with disease outcome (34, 35). Interestingly, another infection-induced anti-inflammatory cytokine transforming growth factor-beta (TGF-β) was not affected upon IL-33 neutralization, suggesting that TGF-β may not be under the control of the IL-33 pathway (Fig. 1*G*). Since IL-10 is known to suppress proinflammatory cytokines by compromising T-cell activation or antigen-presenting cell’s function, therefore, we determined the level of TNF-α and IL-12 secretion from infected cells in the absence of IL-10. Administration of IL-10 neutralizing antibody (10 ng/ml) significantly induced TNF-α and IL-12 production, thus indicating IL-33–mediated reduction of inflammatory cytokines could be a consequence of increased IL-10 production (Fig. S1*C*). All these observations altogether suggest that *Leishmania-*induced IL-33 plays a critical role in parasite survival and shifts host cytokine balance in favor of the parasites.

### Role of IL-33 in the progression of VL in mice

**Figure 2:**
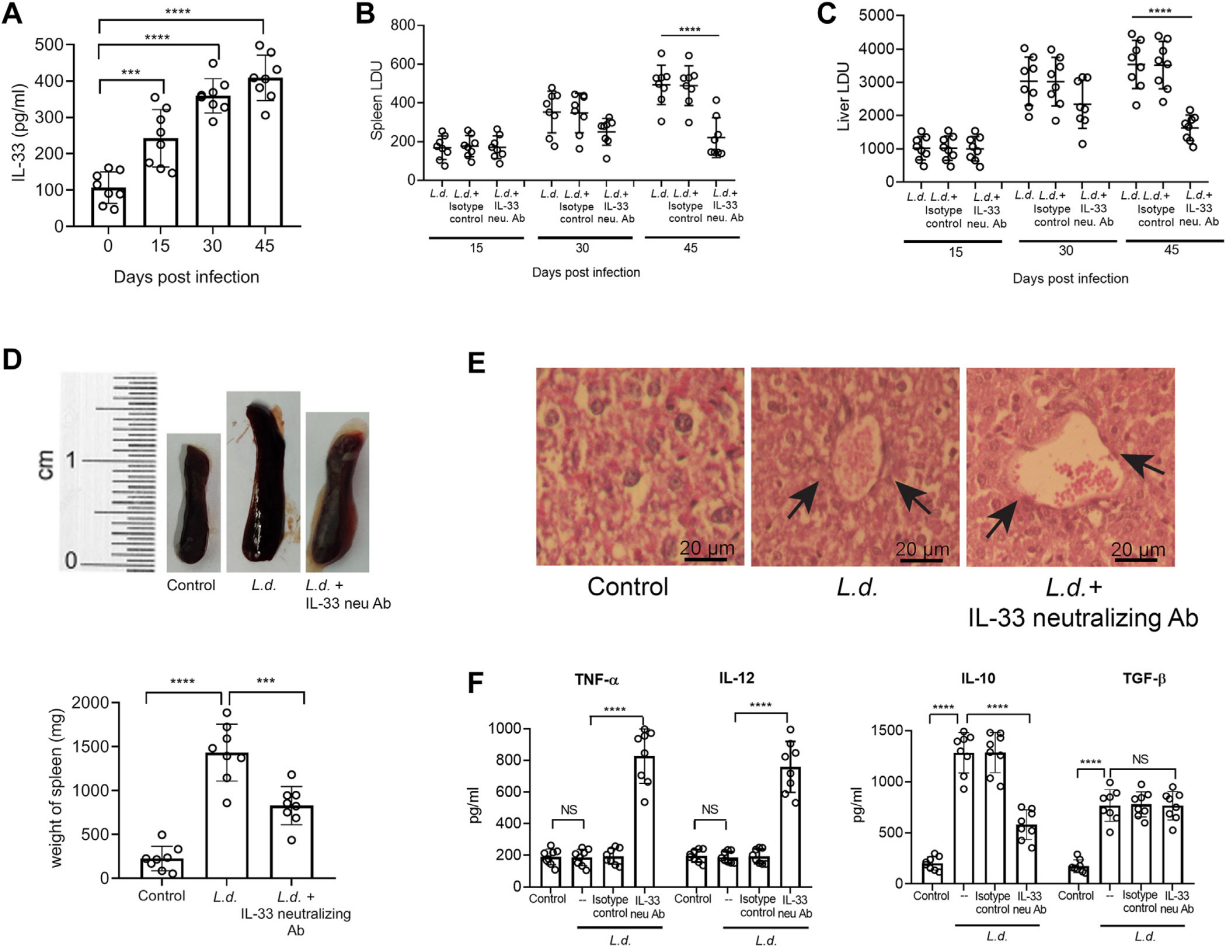
**IL-33 influences visceral infection in BALB/c mice.***A*, BALB/c mice were infected with *Leishmania donovani* promastigotes, and the splenocytes were isolated after indicated periods. IL-33 level was evaluated by ELISA (n = 8). *B* and *C*, *L. donovani*–infected BALB/c mice were treated with anti-IL-33 antibody (50 μg per day at 3 days intervals until sacrifice). Immunoglobulin G was used as an isotype control. The spleen and liver parasite burdens were then measured after the indicated periods and expressed as Leishman–donovan units (n = 8). *D*, spleens were isolated from control, infected, and infected plus anti-IL-33 antibody–treated mice at 45 days postinfection and spleen weight was measured (n = 8). *E*, representative microscopic images of H&E-stained liver sections of different groups are shown (n = 8). *F*, culture supernatant of splenocytes from control, infected, and infected plus anti-IL-33 antibody–treated mice at 45 days postinfection were assayed for TNF-α, IL-12, IL-10, and TGF-β by ELISA (n = 8). The *graph* shows the combined (mean) outcomes from the indicated number of independent experiments, and the error bars indicate the variation between those independent repeats (mean ± SD); NS, not significant, ∗*p* < 0.05, ∗∗*p* < 0.01, ∗∗∗*p* < 0.001, ∗∗∗∗*p* < 0.0001 (ANOVA with Tukey *post hoc* test). IL, interleukin; TGF-β, transforming growth factor-beta.

We next determined the involvement of IL-33 in the progression of VL in the *in vivo* mouse model. Similar to *in vitro* scenario, increasing levels of IL-33 were observed in the culture supernatants of splenocytes isolated from infected mice at 15-, 30-, and 45-days postinfection with a maximum level at 45 days postinfection (3.8-fold over uninfected mice, *p* < 0.0001, F _(3, 28)_ = 40.77) (Fig. 2*A*). Administration of anti-IL-33 antibody (50 μg per day at 3 days interval until sacrifice) significantly reduced the spleen and liver parasite burden (55.2% and 53.9%, respectively, *p* < 0.0001, F _(2, 21)_ = 18.59 and *p* < 0.0001, F _(2, 21)_ = 24.39) (Fig. 2, *B* and *C*). Marked reduction in mean spleen weight was also observed in anti-IL-33 antibody–treated mice (42.1%, *p* < 0.0001, F _(2, 21)_ = 50.67) compared with infected ones (Fig. 2*D*). H&E staining of the liver sections of infected BALB/c mice at 45 days postinfection revealed barely detectable granuloma formation. In contrast, photomicrographs from IL-33 antibody–treated infected mice documented a well-defined zone of granuloma formation (Fig. 2*E*). The levels of the proinflammatory and anti-inflammatory cytokines were next evaluated in the culture supernatant of splenocytes from control, infected, and anti-IL-33 antibody–treated infected mice at 45 days postinfection. IL-33 antibody administration resulted in a marked increase in the level of TNF-α and IL-12 compared with the infected control (4.5-fold and 4.1-fold, respectively, *p* < 0.0001, F _(3, 28)_ = 88.55 and *p* < 0.0001, F _(3, 28)_ = 83.34, respectively). In contrast, treatment with an anti-IL-33 antibody resulted in a significant reduction in IL-10 level compared with untreated infected mice (55.1%, *p* < 0.0001, F _(3, 28)_ = 89.61). But no change was observed in the production of TGF-β (Fig. 2*F*), thereby implying that IL-33–mediated immunosuppression might not involve TGF-β. All these results collectively demonstrate that *Leishmania* infection is associated with the induction of IL-33, which might be exploited as a therapeutic lead.

### *L. donovani* activates the cAMP–EPAC–Rap1 pathway for IL-33 activation

**Figure 3:**
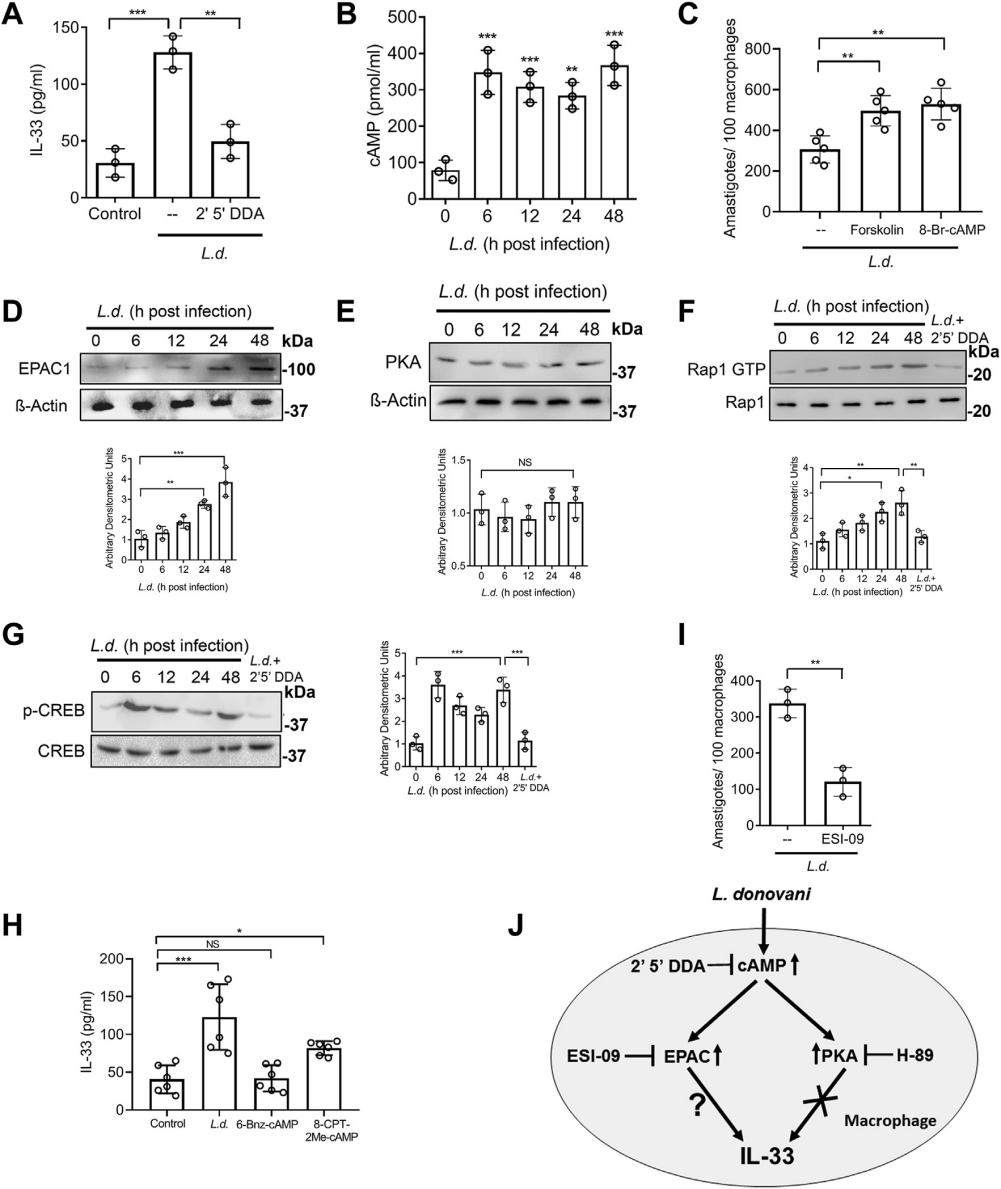
***Leishmania donovani* activates the cAMP–EPAC–Rap1 pathway for increased production of IL-33.***A*, RAW 264.7 cells were infected with *L. donovani* promastigotes pretreated or not with DDA (100 μM). Expression of IL-33 was measured at the protein level by ELISA after 48 h of infection (n = 3). *B*, intracellular cAMP level was determined for indicated periods of infection (n = 3). Statistical significance has been marked relative to uninfected control. *C*, macrophages were infected with *L. donovani* promastigotes for 48 h pretreated with either forskolin (10 μM) or 8-Br-cAMP (100 μM), and the number of amastigotes per 100 macrophages was determined by PI staining (n = 5). *D* and *E*, RAW 264.7 cells were infected with promastigotes, and the protein level expression of EPAC1 and PKA was measured by immunoblotting (n = 3). Statistical significance has been marked relative to uninfected control. *F* and *G*, macrophages were infected with *L. donovani* promastigotes for indicated periods (0–48 h) pretreated or not with DDA and the level of intracellular Rap1-GTP and phospho-CREB were estimated by immunoblotting (n = 3). *H*, macrophages were treated with either PKA agonist or EPAC agonist and the culture supernatants were assayed for IL-33 by ELISA (n = 6). *I*, macrophages were treated with ESI-09, followed by infection with *L. donovani* promastigotes and the intracellular parasite number was determined at 48 h postinfection by PI staining (n = 3). *J*, schematic representation of IL-33 production following infection. The *graph* shows the combined (mean) outcomes from the indicated number of independent experiments, and the error bars indicate the variation between those independent repeats (mean ± SD); NS, not significant, ∗*p* < 0.05, ∗∗*p* < 0.01, ∗∗∗*p* < 0.001, ∗∗∗∗*p* < 0.0001 (Student’s *t* test and ANOVA with Tukey post hoc test). CREB, cAMP-response element-binding protein; DDA, DDA, 2′, 5′-dideoxyadenosine; EPAC, exchange protein activated by cAMP; IL, interleukin; PI, propidium iodide.

We next sought to determine the underlying mechanism behind the enhanced expression of IL-33. cAMP has been shown to induce IL-33 production in BMDM (29). The role of cAMP was, therefore, checked in infection-induced IL-33 production by using 2′, 5′-dideoxyadenosine (DDA), the specific inhibitor of adenylate cyclase, (28). When cells were treated with DDA (100 μM for 1 h) followed by infection for 48 h, the level of IL-33 was decreased as compared to infected control (61.2%, *p* = 0.000330, F _(2, 6)_ = 40.42) (Fig. 3*A*). Intracellular cAMP level [cAMP]i also was found to be increased at 48 h postinfection (a 4.6-fold increase compared to uninfected cells (*p* = 0.000119, F _(4, 10)_ = 18.84) (Fig. 3*B*). The role of [cAMP]i on parasite survival was then determined by pretreating infected macrophages with 10 μM (for 1 h) forskolin (activator of adenylyl cyclase) and 100 μM (for 1 h) 8-Br-cAMP (cell-permeable cAMP analog), which significantly increased the intramacrophage survival of parasite (1.6- and 1.7-fold, respectively, *p* = 0.000855, F _(2, 12)_ = 13.47) (Fig. 3*C*). In order to find out whether cAMP-mediated parasite survival is absolutely dependent on IL-33, infected macrophages were treated with cAMP inhibitor (2′ 5′ DDA, 100 μM) in the presence or absence of recombinant IL-33 and intracellular parasite number was determined. A significant decrease in parasite survival was observed by treatment with DDA, which was only partially reversed by the addition of recombinant IL-33 (Fig. S2*A*), suggesting that IL-33 signaling might be one of the several pathways regulated by cAMP during infection. Since the effects of cAMP signaling are attributed to its downstream effector molecules PKA and EPAC (36, 37), we next checked their expression in infected RAW and BMDM (Figs. 3*D* and S2*B*). Time kinetics study revealed increased expression of EPAC1 in infected cells as compared with uninfected control with a maximum increase at 48 h postinfection (3.7-fold, *p* = 0.000129, F _(4, 10)_ = 18.50) (Fig. 3*D*). EPAC2 was not evaluated as its expression was reported to be negligible in RAW 264.7 cells (22). However, *L. donovani* infection did not affect the expression of PKA in macrophages (Fig. 3*E*). In addition to expression, we also checked the activity of EPAC1 and PKA after infection. As the principal function of EPAC is to act as a guanine nucleotide exchange factor (GEF) for Rap GTPases and activate those by transferring GTP (38), therefore Rap1-GTP level was measured in cell lysates of infected macrophages at various time points postinfection. Similar to EPAC1 expression, the level of Rap1-GTP also showed an increasing pattern as observed up to 48 h postinfection (a 2.4-fold increase compared to uninfected control, *p* = 0.001085, F _(5, 12)_ = 8.73) (Fig. 3*F*). To determine whether infection induces PKA activation, we checked the phosphorylation of specific PKA substrate cAMP-response element-binding protein (CREB). Unlike PKA expression, phospho-CREB showed a significant increase over the uninfected control (Fig. 3*G*). Activation of both EPAC and PKA was inhibited when infected macrophages were pretreated with DDA (Fig. 3, *F* and *G*). To assess the role of PKA and EPAC in infection-induced IL-33 production, infected macrophages were further pretreated with either PKA inhibitor H-89 (28) or EPAC inhibitor ESI-09 (39), and IL-33 level was determined in the culture supernatant. Interestingly, IL-33 secretion from infected cells remained almost unaltered when pretreated with H-89 (10 μM for 1 h), whereas pretreatment with ESI-09 (10 μM for 1 h) markedly reduced IL-33 secretion (Fig. S2*C*), thus indicating EPAC as the key player in cAMP-mediated IL-33 production. A similar pattern was also observed in BMDM (Fig. S2*D*). To further ascertain the exclusive involvement of EPAC in the regulation of IL-33, we treated control macrophages with PKA and EPAC-specific agonists for 24 h (6-Bnz-cAMP and 8-CPT-2Me-cAMP, respectively) and measured IL-33 levels. We found that PKA agonist 6-Bnz-cAMP (50 μM) did not exert any noticeable effect on IL-33 production. In contrast, EPAC agonist 8-CPT-2Me-cAMP (50 μM) resulted in increased expression of IL-33 (1.8-fold as compared to control cells (*p* < 0.0001, F _(3, 20)_ =14.08) (Fig. 3*H*). This moderate elevation of IL-33 in the presence of EPAC agonist is may be due to low expression of EPAC in uninfected macrophages. To further ascertain the exclusive involvement of EPAC in the regulation of IL-33, we knocked down PKA and EPAC by siRNA-mediated gene silencing in infected macrophages and measured IL-33 levels. siRNA efficiency was evaluated by immunoblot analysis. EPAC-silenced infected cells exhibited markedly reduced expression of IL-33 at 48 h postinfection (Fig. S2*E*). On the contrary, the silencing of PKA did not significantly influence infection-induced IL-33 expression (Fig. S2*E*). Moreover, the administration of ESI-09 significantly reduced intracellular parasite burden (64.2% compared to infected control, *p* = 0.0026) (Fig. 3*I*). Taken together, these results suggest that PKA and EPAC although activated by infection, which do not act synergistically. Moreover, induction of IL-33 during infection is under the exclusive control of EPAC (Fig. 3*J*). No cytotoxic or cytostatic effect was observed for all the compounds used in the above-mentioned experiments as assessed by cell viability using an 3-(4,5-dimethylthiazol-2-yl)-2,5-diphenyltetrazolium bromide (MTT)-based colorimetric assay (Fig. S2, *F* and *G*).

### EPAC signaling–induced intracellular calcium is crucial in IL-33 production

**Figure 4:**
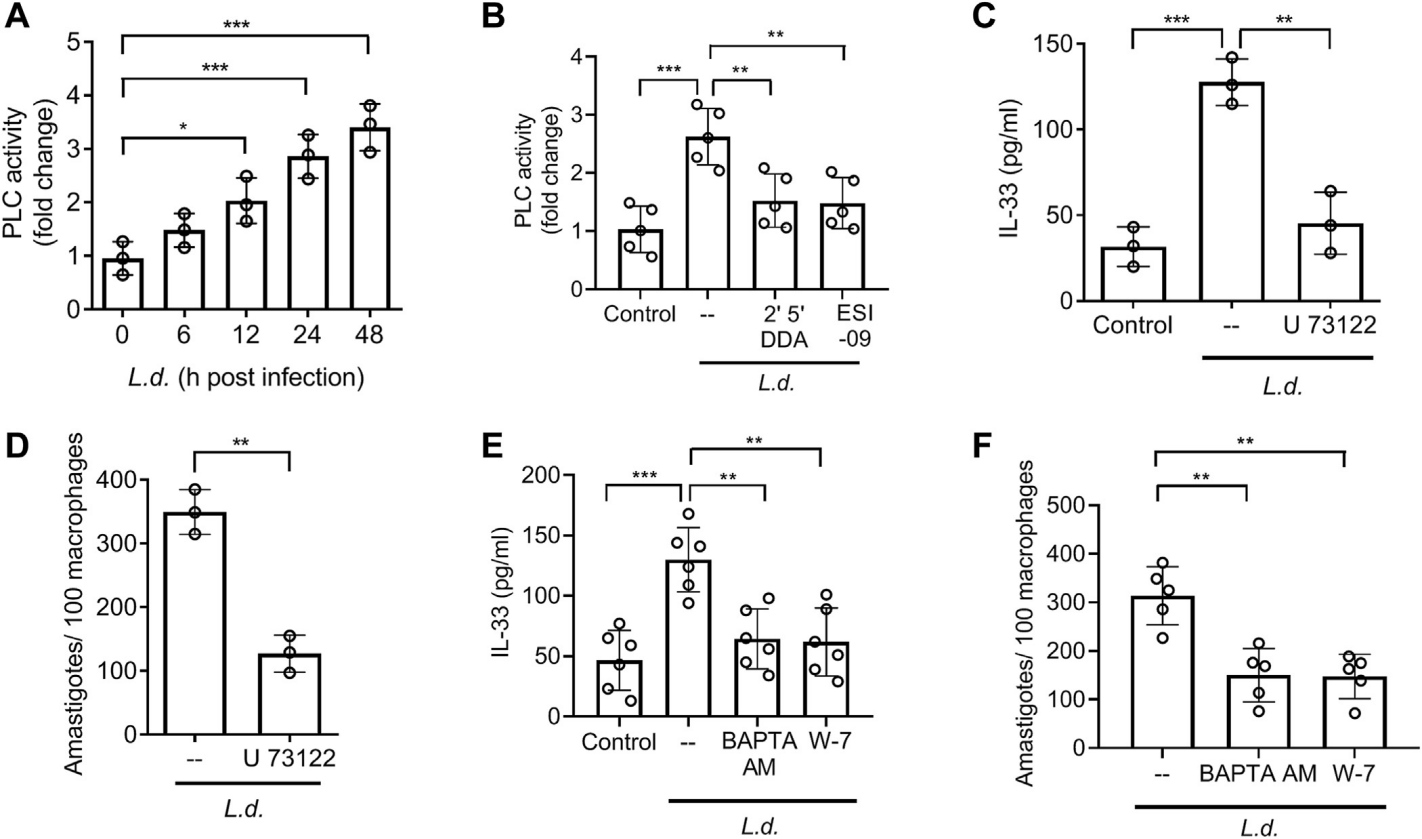
**Role of EPAC-mediated induction of intracellular calcium in IL-33 production.***A*, RAW 264.7 cells were infected with *Leishmania donovani* promastigotes for indicated periods and the activity of PLC was measured (n = 3). Statistical significance has been marked relative to uninfected control. *B*, PLC activity was estimated in 48 h infected macrophages pretreated with either DDA (100 μM) or ESI-09 (10 μM) (n = 5). *C*, infected RAW 264.7 cells were pretreated with U 73122 (10 μM), and IL-33 levels were measured at 48 h postinfection (n = 3). *D*, infected (48 h) macrophages were pretreated with U 73122 (10 μM), and the number of parasites per 100 macrophages was determined by PI staining (n = 3). *E*, infected macrophages were preincubated with BAPTA AM (10 μM) or W-7 hydrochloride (10 μM), and the levels of IL-33 were estimated by ELISA (n = 6). *F*, intracellular parasite number was determined in BAPTA AM (10 μM) and W-7 hydrochloride (10 μM) pretreated macrophages (n = 5). The *graph* shows the combined (mean) outcomes from indicated number of independent experiments, and the error bars indicate the variation between those independent repeats (mean ± SD); NS, not significant, ∗*p* < 0.05, ∗∗*p* < 0.01, ∗∗∗*p* < 0.001, ∗∗∗∗*p* < 0.0001 (Student’s *t* test and ANOVA with Tukey *post hoc* test). BAPTA AM, 1,2-Bis(2-aminophenoxy)ethane-N,N,N',N'-tetraacetic acid tetrakis(acetoxymethyl ester); DDA, 2′, 5′-dideoxyadenosine; EPAC, exchange protein activated by cAMP; IL, interleukin; PI, propidium iodide; PLC, phospholipase C.

Since PLC is the well-characterized component of EPAC signaling (40, 41), we, therefore, measured the activity of PLC at various time points after *L. donovani* infection. Infected macrophages showed increased PLC activity with maximum activity at 48 h postinfection (3.5-fold increase compared to uninfected control, *p* < 0.0001, F _(4, 10)_ = 20.19) (Fig. 4*A*). This increased PLC activity was inhibited when cells were preincubated with either DDA or ESI-09 (42.3% and 46.2% decrease, respectively, compared to infected control, *p* = 0.000285, F _(3, 16)_ = 11.52) (Fig. 4*B*). To ascertain further the role of PLC in IL-33 production, IL-33 secretion from infected cells was measured after pretreatment with the PLC inhibitor U 73122 (10 μM for 1 h) (42), which inhibited IL-33 production in RAW (64.5% decrease compared to infected control, *p* = 0.000397, F _(2, 6)_ = 37.81) (Fig. 4*C*) and BMDM (Fig. S3*A*). Administration of U 73122 also decreased the number of parasites in infected cells (63.7% decrease compared to infected control, *p* = 0.0011) (Fig. 4*D*). Interestingly, mRNA expression of all the PLC isoforms did not document any appreciable change during infection (Fig. S3*B*), suggesting posttranscriptional control of PLC in infection. Since PLC activation is intricately associated with intracellular calcium release (43, 44), we next checked whether calcium is involved in infection-mediated IL-33 production. Therefore, infected macrophages were preincubated with cell-permeable calcium ion chelator 1,2-Bis(2-aminophenoxy)ethane-N,N,N',N'-tetraacetic acid tetrakis(acetoxymethyl ester) (BAPTA AM) (10 μM for 1 h) (45) and measured the level of secreted IL-33, which was significantly decreased (50.8% decrease compared to the infected control) (Fig. 4*E*). In eukaryotic cells, the primary intracellular receptor for calcium is calmodulin (46, 47). To determine whether calcium-induced IL-33 is also regulated by the calcium/calmodulin complex, we then employed the calmodulin inhibitor W-7 hydrochloride (48) while measuring IL-33 secretion from infected cells. The increase in IL-33 production was reversed when infected macrophages were pretreated with 10 μM W-7 hydrochloride for 1 h (53.1% decrease compared to infected cells, *p* = 0.000102, F _(3, 20)_ = 12.01) (Fig. 4*E*). Similar trend was obtained in BMDM (Fig. S3*C*). Treatment with BAPTA AM and W-7 hydrochloride also significantly reduced intracellular parasite burden (52.1% and 52.9%, respectively, *p* = 0.000466, F _(2, 12)_ = 15.55) (Fig. 4*F*). There was no cytotoxic effect of the compounds used in the above experiments (Fig. S3*D*). All these observations strongly suggest that infection-induced EPAC activates the PLC/calcium/calmodulin axis leading to IL-33 production.

### Essential role of calcium-stimulated calcineurin activity in IL-33 generation

**Figure 5:**
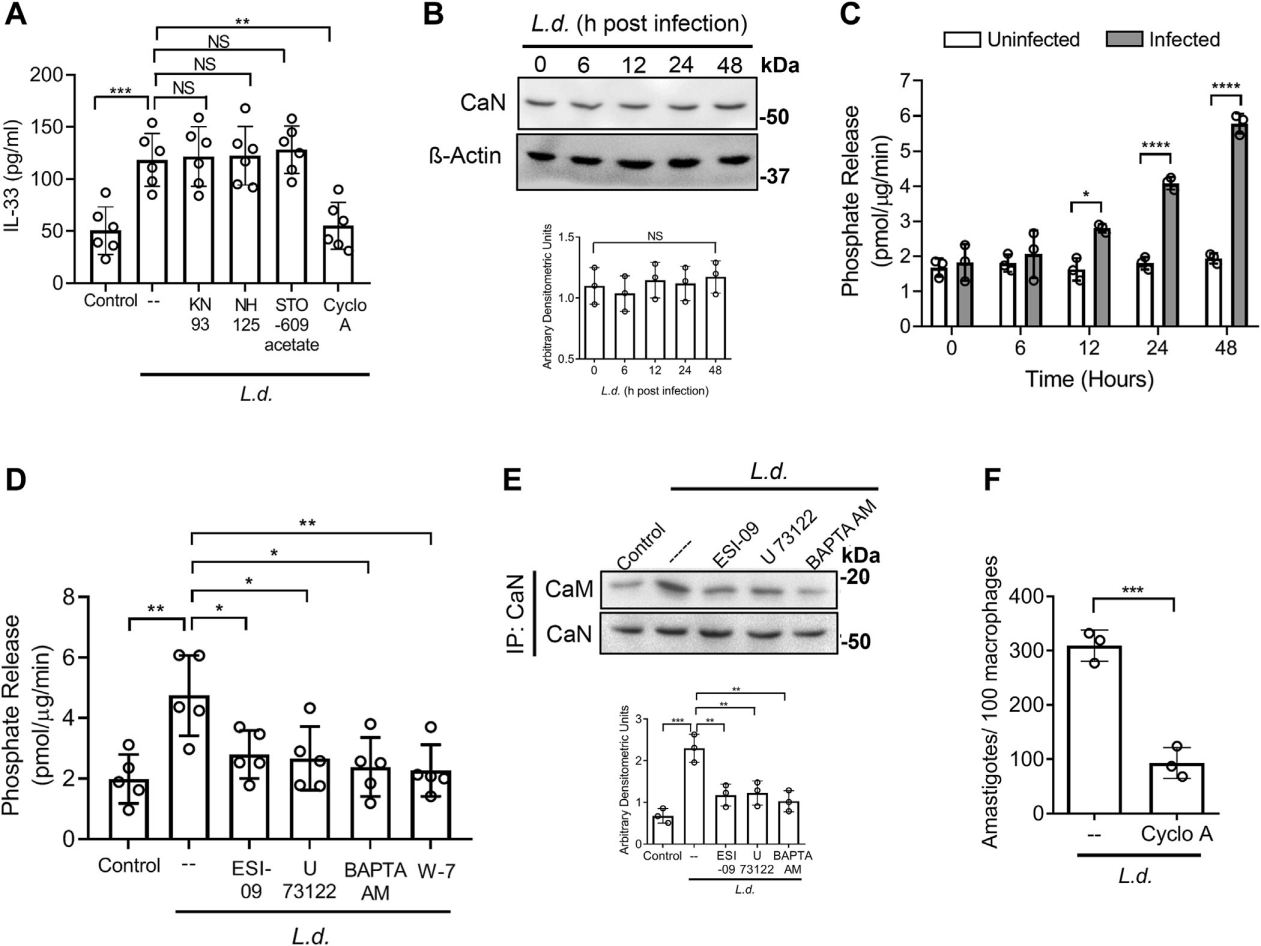
**Essential role of calcium-stimulated calcineurin activity in IL-33 generation.***A*, RAW 264.7 cells were treated with KN93 (1 μM), NH 125 (3 μM), STO-609 acetate (10 μM), and cyclosporin A (1 μg/ml) before infection with *Leishmania donovani* promastigotes, and IL-33 levels were measured (n = 6). *B*, cells were infected for indicated periods, and the protein level expression of calcineurin was assessed by immunoblotting. β-Actin was used as an endogenous control (n = 3). *C*, calcineurin activity was measured in RAW 264.7 cells following *L. donovani* infection (0–48 h) (n = 3). *D*, calcineurin activity was assayed in infected macrophages (48 h) pretreated with either ESI-09 or U 73122 or BAPTA AM or W-7 hydrochloride (n = 5). *E*, macrophages were infected with *L. donovani* pretreated or not with either ESI-09 or U 73122 or BAPTA-AM. Whole-cell lysates were subjected to immunoprecipitation with an anti-calcineurin antibody. Immunoprecipitates were then subjected to Western blotting with an anti-calmodulin antibody (n = 3). *F*, cells were infected for 48 h with *L. donovani* pretreated or not with cyclosporin A, and intracellular parasite numbers were determined by PI staining (n = 3). The *graph* shows the combined (mean) outcomes from the indicated number of independent experiments, and the error bars indicate the variation between those independent repeats (mean ± SD); NS, not significant, ∗*p* < 0.05, ∗∗*p* < 0.01, ∗∗∗*p* < 0.001, ∗∗∗∗*p* < 0.0001 (Student’s *t* test and ANOVA with Tukey post hoc test). BAPTA AM, 1,2-Bis(2-aminophenoxy)ethane-N,N,N',N'-tetraacetic acid tetrakis(acetoxymethyl ester); IL, interleukin; PI, propidium iodide.

Now, to delineate the precise mechanism of calcium-calmodulin–regulated IL-33 production in infection, an inhibitor-based approach was taken. IL-33 secretion was measured in *L. donovani*–infected RAW 264.7 (Fig. 5*A*) and BMDM (Fig. S4*A*) pretreated with various inhibitors of calcium-calmodulin–dependent enzymes. KN93 (1 μM for 1 h) for CaM kinase II (49), NH 125 (3 μM for 1 h) for CaM kinase III (50), STO-609 acetate (10 μM for 1 h) for CaM kinase kinase (51), and cyclosporin A (1 μg/ml for 1 h) for calcineurin (52) were used. Of the inhibitors, only cyclosporine A exerted a significant effect on IL-33 secretion (53.6% decrease compared to infected control, *p* < 0.0001, F _(5, 30)_ = 12.48) (Fig. 5*A*). Increasing the concentration of other kinase inhibitors did not influence IL-33 level, implicating calcineurin to be the major regulator of IL-33 (data not shown). Although no significant change in expression of calcineurin in infection was detected as observed up to 48 h (Fig. 5*B*), activity of calcineurin was increased markedly with maximum activity (2.9-fold compared to the uninfected cells, *p* < 0.0001, F _(4, 20)_ = 31.28) at 48 h postinfection in case of RAW 264.7 cells (Fig. 5*C*). Similar results were also obtained for BMDM (Fig. S4*B*). Elevated calcineurin activity was inhibited when infected cells were pretreated with ESI-09 (EPAC inhibitor, 10 μM) (41.1% decrease compared to the infected control), U 73122 (PLC inhibitor, 10 μM) (43.7%, decrease compared to the infected control), BAPTA AM (calcium chelator, 10 μM) (49.8% decrease compared to the infected control), and W-7 hydrochloride (calmodulin antagonist, 10 μM) (52.3% decrease compared to the infected control) independently (*p* = 0.002590, F _(5, 24)_ = 5.07) (Fig. 5*D*), validating the EPAC/PLC/calcium/calmodulin signaling axis in calcineurin activation. We further checked the interaction of calmodulin and calcineurin in infected cells and similar to calcineurin activity, the interaction between calcineurin and calmodulin was partially restricted in infected cells pretreated with ESI-09 (48.8% decrease compared to the infected control), U 73122 (46.7% decrease compared to the infected control) and BAPTA AM (55.2% decrease compared to the infected control), respectively (*p* = 0.000283, F _(4, 10)_ = 15.39) (Fig. 5*E*). It is possible that EPAC/PLC-independent calcium release may be also induced by infection, however, the magnitude is too little to contribute significantly to IL-33 production. To determine whether activated calcineurin aids in parasite survival, macrophages were treated with cyclosporin A before parasite infection. A significant decrease in intracellular parasite burden was obtained upon pretreatment with cyclosporin A (69.9% decrease compared to infected control, *p* = 0.0008) (Fig. 5*F*), further correlating IL-33 level with parasite survival. No cytotoxic effect was observed for the inhibitors used in the above-mentioned experiments (Fig. S4*C*). Collectively, these results suggest that downstream of PLC, calcium-dependent activation of calcineurin is responsible for infection-mediated induction of IL-33 in macrophages.

### Role of NFATc1 in calcium-dependent transcriptional upregulation of IL-33

**Figure 6:**
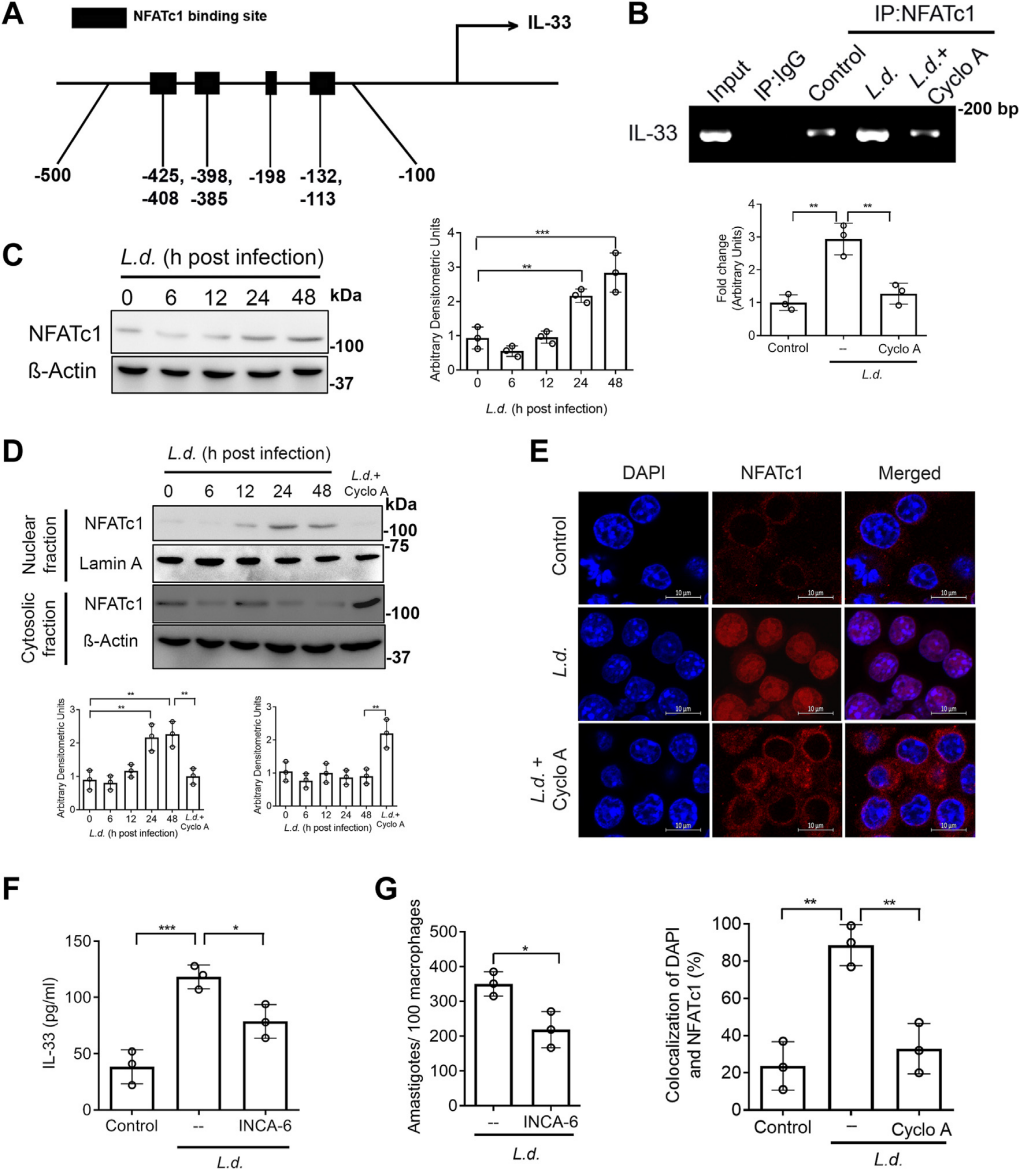
**Role of NFATc1 in calcium dependent transcriptional upregulation of IL-33.***A*, schematic representation of NFATc1 binding site in IL-33 promoter. *B*, DNA from *Leishmania donovani*–infected RAW 264.7 cells were immunoprecipitated with anti-NFATc1 antibody or normal Immunoglobulin G. Immunoprecipitated DNA was then analyzed using IL-33 promoter–specific primers by PCR, followed by agarose gel electrophoresis (n = 3). *C*, RAW cells were infected with *L. donovani* for various periods and expression of NFATc1 was determined by immunoblotting (n = 3). Statistical significance has been marked relative to uninfected control. *D*, macrophages were infected with *L. donovani* promastigotes at indicated periods and the levels of NFATc1 were analyzed in nuclear and cytosolic fractions by immunoblotting (n = 3). *E*, macrophages were infected with *L. donovani* either pretreated or not with cyclosporin A (1 μg/ml) and then stained with anti-NFATc1 antibody, followed by secondary Texas Red–conjugated antibody. Nuclei were stained with DAPI and cells were analyzed under a microscope. Images were analyzed for colocalization using ImageJ software (n = 3). *F* and *G*, RAW cells were treated with INCA-6 (10 μM) before infection, and the level of IL-33 secretion (*F*) and the number of parasites per 100 macrophages (*G*) were determined by ELISA and PI staining, respectively (n = 3). The *graph* shows the combined (mean) outcomes from the indicated number of independent experiments, and the error bars indicate the variation between those independent repeats (mean ± SD); NS, not significant, ∗*p* < 0.05, ∗∗*p* < 0.01, ∗∗∗*p* < 0.001, ∗∗∗∗*p* < 0.0001 (Student’s *t* test and ANOVA with Tukey post hoc test). DAPI, 4′,6-diamidino-2-phenylindole; NFATc, nuclear factor of activated T cell; PI, propidium iodide.

Next, we tried to detect the transcription factors responsible for IL-33 induction during infection. Analysis of the IL-33 promoter region revealed transcription binding sites for NF-κB, CREB, NFATc1, interferon regulatory factor 3 (IRF3), and HIF-1α. Amongst these, we selected NFATc1 (Fig. 6*A*), as it is most well-known to be regulated by calcium (53, 54). Chromatin immunoprecipitation (ChIP) assay indeed documented strong binding of NFATc1 in the IL-33 promoter region (2.9-fold compared to uninfected cells (*p* = 0.001181, F _(2, 6)_ = 25.39) at 48 h postinfection) (Fig. 6*B*). Western blot analysis also showed a significant enhancement in expression of NFATc1 during the late phase of infection with a maximum increase at 48 h postinfection (3.1-fold over uninfected cells, *p* < 0.0001, F _(4, 10)_ = 26.96) (Fig. 6*C*). Nuclear translocation kinetics of NFATc1 in infection was also found to be in synchronization with its expression (2.5-fold over uninfected control, *p* < 0.0001, F _(5, 12)_ = 14.69). This upregulated nuclear translocation of NFATc1 was inhibited when infected cells were pretreated with cyclosporin A, thus indicating the role of calcineurin in this nuclear translocation (Fig. 6*D*). This observation was further verified by microscopic analysis, which also showed enhanced nuclear translocation of NFATc1 following infection (Fig. 6*E*). To ascertain the role of NFATc1 in infection-induced IL-33 production, we measured IL-33 secretion in infected cells pretreated with INCA-6 (10 μM for 1 h), which blocks association between calcineurin and NFATc1 (55). INCA-6 pretreatment significantly reduced IL-33 production in infected cells (33.5% compared to infected control, *p* = 0.001185, F _(2, 6)_ = 25.35) and a similar result was obtained for BMDM (Fig. S5*A*). However, the extent of downregulation was not at par with that obtained when cells were pretreated with inhibitors of signaling compounds upstream of calcineurin (Fig. 6*F*). This finding, therefore, indicates possible involvement of transcription factors other than NFATc1 in infection-induced IL-33 secretion. To determine the role of NFATc1-mediated signaling on parasite survival, infected macrophages were pretreated with INCA-6, and intracellular parasite numbers were measured, which showed a moderate decrease (37.5%, *p* = 0.0222) (Fig. 6*G*). All these observations suggest a critical role of NFATc1 in enhanced IL-33 production. However, it may not be the sole transcription factor for IL-33 production in infection.

### Stabilization and activation of HIF-1α promote IL-33 production during *L. donovani* infection

**Figure 7:**
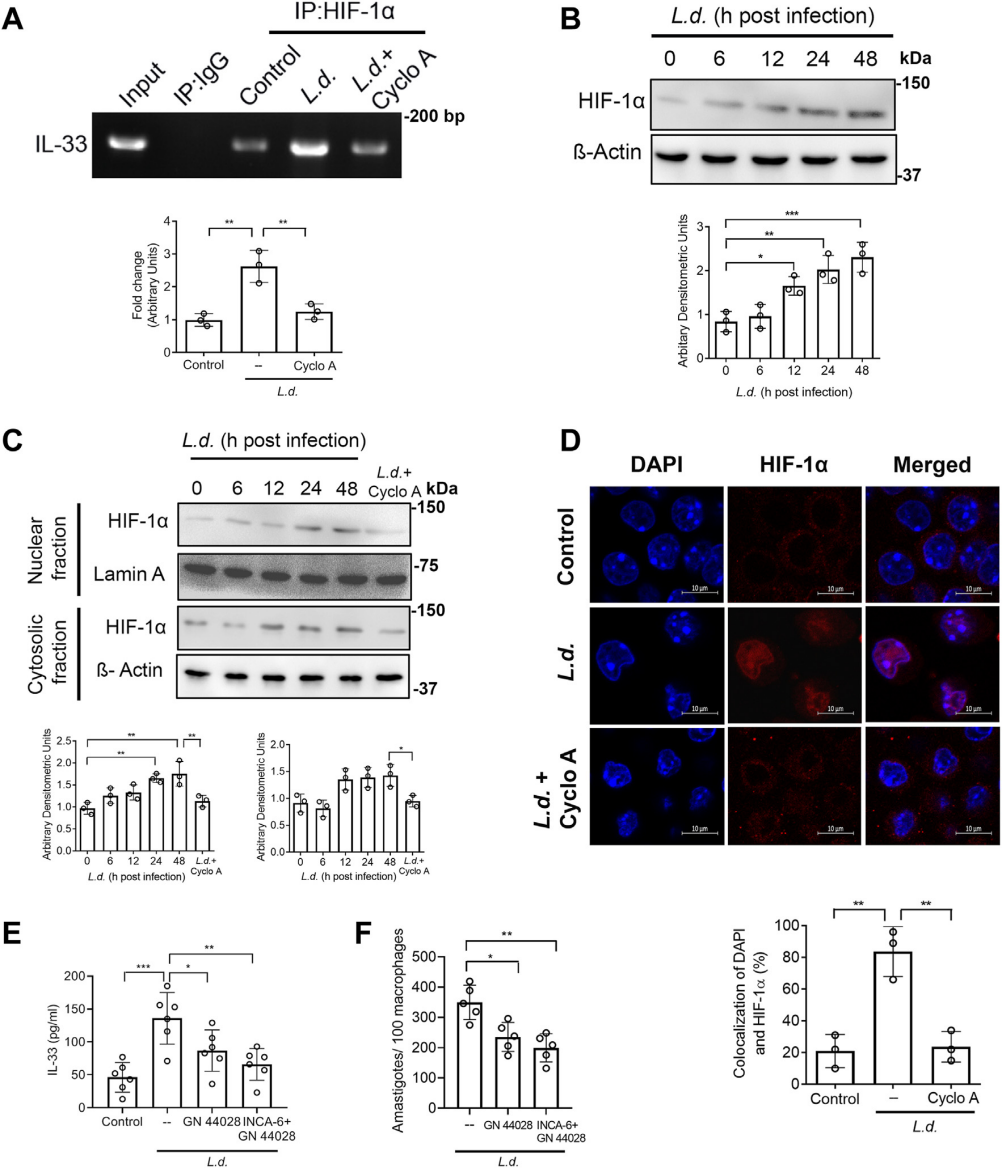
**HIF-1α acts together with NFATc1 for infection-dependent induction of IL-33.***A*, macrophages were infected with *Leishmania donovani* for the indicated periods and analyzed for HIF-1α binding to IL-33 promoter by ChIP assay (n = 3). *B*, cells were infected with *L. donovani* promastigotes for indicated periods (0–48 h), and the protein level expression of HIF-1α was measured by immunoblotting (n = 3). Statistical significance has been marked relative to uninfected control. *C*, Cells were infected with *L. donovani* promastigotes for 0 to 48 h. Nuclear and cytosolic fractions were separated and expression of HIF-1α was evaluated by immunoblotting (n = 3). *D*, cells were infected with promastigotes for 48 h either preincubated or not with cyclosporin A (1 μg/ml) and then stained with HIF-1α antibody, followed by Texas Red–conjugated secondary antibody. Nuclei were stained with DAPI and the cells were analyzed under a confocal microscope; the intensity of colocalization was measured using ImageJ software (n = 3). *E*, macrophages were pretreated either with GN44028 (10 μM) alone or in combination with INCA-6 (10 μM), followed by infection with *L. donovani* for 48 h and level of IL-33 was measured (n = 6). *F*, intracellular parasite numbers were evaluated in infected RAW 264.7 cells pretreated with either GN44028 or a combination of both GN44028 and INCA-6 (n = 5). The *graph* shows the combined (mean) outcomes from the indicated number of independent experiments, and the error bars indicate the variation between those independent repeats (mean ± SD); NS, not significant, ∗*p* < 0.05, ∗∗*p* < 0.01, ∗∗∗*p* < 0.001, ∗∗∗∗*p* < 0.0001 (ANOVA with Tukey post hoc test). ChIP, chromatin immunoprecipitation; DAPI, 4′,6-diamidino-2-phenylindole; HIF-1α, hypoxia-inducible factor 1 alpha; NFATc, nuclear factor of activated T cell; PI, propidium iodide.

As previous observation indicated the involvement of more calcineurin-dependent transcription factors in IL-33 induction, we next reviewed the role of HIF-1α in the production of IL-33 (56). Nuclear translocation of both NF-κB and IRF3 are known to be impaired during *L. donovani* infection (34) and CREB is reported to be primarily regulated by PKA (57), therefore we focused on HIF-1α. However, CREB can also be regulated downstream of Ca^2+^ signaling, either by phosphorylation or *via* its coactivators CRTC. Therefore, we inhibited CREB by its specific inhibitor 666-15 (5 μM) in infected macrophages and measured the IL-33 level (Fig. S5*B*). Comparable levels of IL-33 were obtained in both infected cells and inhibitor-treated infected cells, suggesting thereby that CREB may not be involved in the regulation of IL-33. We examined the effect of *L. donovani* infection on the binding of HIF-1α to IL-33 promoter regions by ChIP assay and found a marked increase in binding (2.6-fold over uninfected control, *p* = 0.002007, F _(2, 6)_ = 20.78) (Fig. 7*A*). However, as far as expression is concerned, infection led to significant enhancement in HIF-1α expression over control cells during late hours of infection (2.8-fold at 48 h postinfection, *p* = 0.000246, F _(4, 10)_ = 15.90) (Fig. 7*B*). Immunoblot analysis also revealed an increase in nuclear HIF-1α in infected macrophages (1.8-fold over uninfected control, *p* = 0.000997, F _(5, 12)_ = 8.89). Similar to NFATc1, cyclosporin A (1 μg/ml for 1 h) pretreatment also reduced the nuclear localization of HIF-1α. However, unlike NFATc1, cytosolic HIF-1α was also decreased in case of pretreatment of cyclosporin A, thus indicating a role of calcineurin in the stabilization of HIF-1α following infection (Fig. 7*C*). Microscopic analysis also confirmed this observation (Fig. 7*D*). To ascertain the role of HIF-1α, IL-33 secretion from infected cells were evaluated in the presence of GN44028 (10 μM for 1 h), a transcriptional inhibitor of HIF-1α (58), which resulted in a moderate decrease in IL-33 production (36.2% reduction as compared to infected control). However, A marked reduction was obtained in RAW 264.7 (51.6% as compared to the infected control, *p* = 0.000333, F _(3, 20)_ = 9.87) (Fig. 7*E*), and BMDM (Fig. S5*C*) when inhibitors of both NFATc1 (INCA-6, 10 μM for 1 h) and HIF-1α (GN44028, 10 μM for 1 h) were used in combination, suggesting a synergistic effect of both NFATc1 and HIF-1α in infection mediated IL-33 induction. The inhibitory effect on intracellular parasite burden was also much greater when GN44028 and INCA-6 were used in combination than the use of GN44028 alone (Fig. 7*F*). None of the inhibitors had any effect on cell viability (Fig. S5*D*). Taken together, these results indicate a synergistic effect of NFATc1 and HIF-1α in the transcriptional upregulation of IL-33 in macrophage during *L. donovani* infection.

### NFATc1- and HIF-1α–dependent IL-33 production aids in parasite survival in mouse models of VL

**Figure 8:**
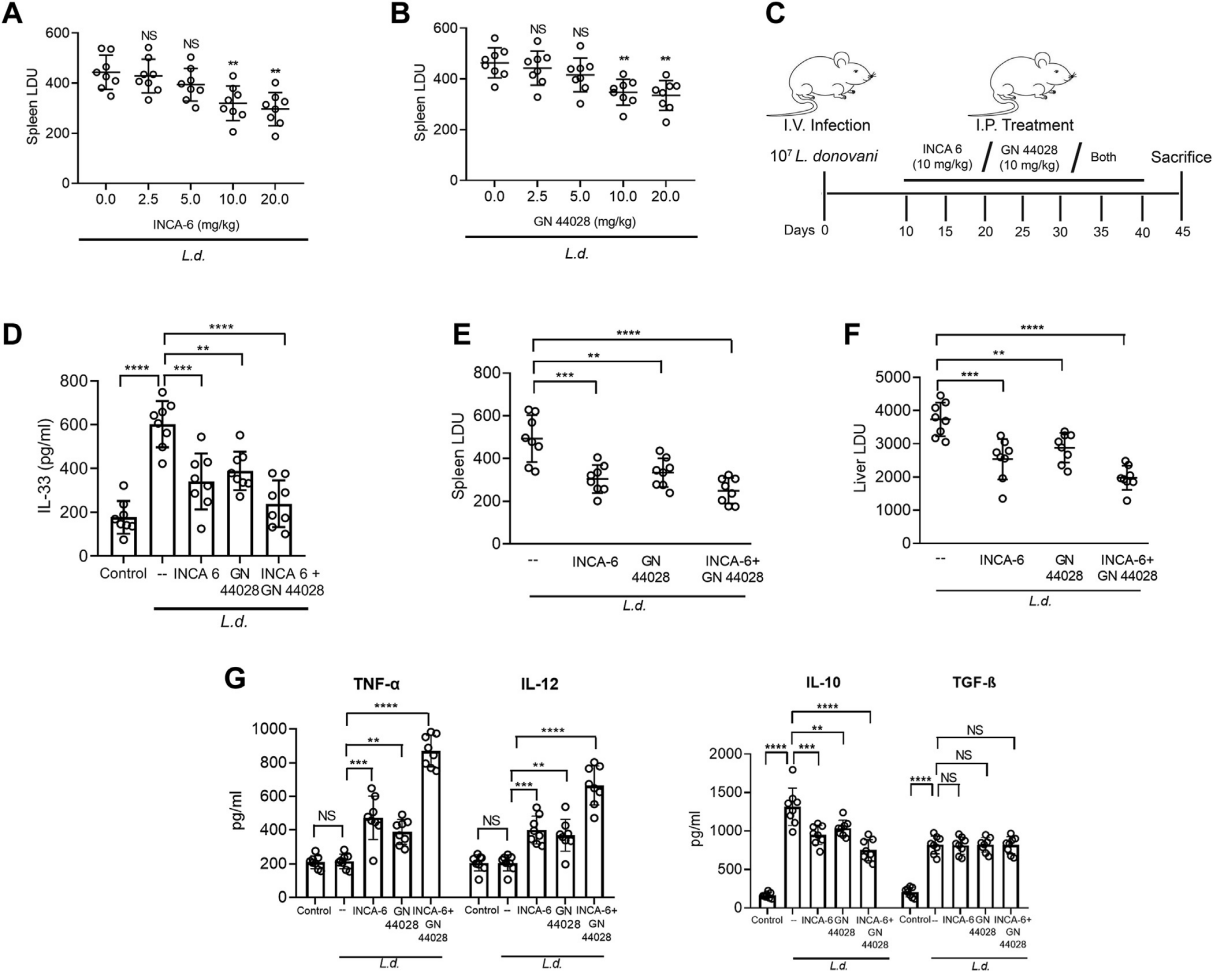
**NFATc1 and HIF-1α are responsible for IL-33 production in mouse model of visceral leishmaniasis.***A* and *B*, various doses of INCA-6 and GN 44028 ranging from 2.5 to 20 mg/kg body weight/day were given i.p. up to 45 days at every 5 days starting from the 10th day postinfection. The splenic parasite burden were then determined for both the inhibitors (n = 8). *C*, the course of infection was followed in *Leishmania donovani*–infected BALB/c mice that had received i.p. injections of INCA-6 (10 mg/kg body weight/day) and GN44028 (10 mg/kg body weight/day) for 45 days at 5 days interval starting after 10 days of infection. *D*, the level of IL-33 in the culture supernatant of isolated splenocytes was measured by ELISA in various treated groups 45 days after infection (n = 8). *E* and *F*, spleen and liver LDU were measured in various treated groups 45 days after infection (n = 8). *G*, at 45 days postinfection, culture supernatant of splenocytes from control, infected, and infected mice treated with INCA-6 and GN 44028 either independently or in combination were assayed for TNF-α, IL-12, IL-10, and TGF-β by ELISA (n = 8). The *graph* shows the combined (mean) outcomes from the indicated number of independent experiments, and the error bars indicate the variation between those independent repeats (mean ± SD); NS, not significant, ∗*p* < 0.05, ∗∗*p* < 0.01, ∗∗∗*p* < 0.001, ∗∗∗∗*p* < 0.0001 (ANOVA with Tukey post hoc test). HIF-1α, hypoxia-inducible factor 1 alpha; IL, interleukin; i.p. intraperitoneally; LDU, Leishman-Donovan units; NFATc, nuclear factor of activated T cell; PI, propidium iodide; TGF-β, transforming growth factor-beta.

To investigate whether *L. donovani*–induced IL-33 signaling is also operative in the *in vivo* situation, infected mice were administered intraperitoneally either with INCA-6 or GN44028 with different doses (2.5, 5, 10, and 20 mg/kg body weight/day given up to 45 days at every 5 days starting at the 10th day postinfection) and spleen parasite burden was measured at 45 days after infection. Maximum suppression of parasite burden was obtained at a dose of 10 mg/kg body weight for both the inhibitors (Fig. 8, *A* and *B*) without causing any apparent change in the pathophysiology of the mice and therefore this dose was chosen for the subsequent experiments. *L. donovani–*infected mice were then administered with INCA-6 and GN44028 independently or in combination at a dose of 10 mg/kg body weight/day up to 45 days at every fifth day starting from 10th day postinfection (Fig. 8*C*), and IL-33 level was determined in the isolated splenocytes of infected and treated animals. Infection-mediated upregulation of IL-33 was markedly inhibited when INCA-6 and GN 44028 were administrated individually (43.5% and 35.4% inhibition, respectively). However, simultaneous administration of these two compounds reduced IL-33 level much more significantly (60.1% decrease as compared to infected control, *p* < 0.0001, F _(4, 35)_ = 20.69) (Fig. 8*D*). To determine the role of IL-33 on parasite survival, we treated infected mice with INCA-6 or GN 44028 or both and spleen and liver parasite burden were assessed by Giemsa staining. Splenic and hepatic parasite burden was reduced in INCA-6– and GN 44028–treated mice (a decrease of 38.3% and 32.3% in spleen, 31.9% and 22.9% in liver, respectively) at 45 days postinfection. This reduction was much more significant when mice were treated simultaneously with both the inhibitors (49.4% reduction in spleen Leishman-Donovan units, *p* < 0.0001, F _(3, 28)_ = 14.32, and 47.1% reduction in liver Leishman-Donovan units, *p* < 0.0001, F _(3, 28)_ = 18.03) (Fig. 8, *E* and *F*). Independent treatment of INCA-6 increased TNF-α (2.2-fold) and IL-12 level (1.9-fold) and that of GN 44028 increased TNF-α (1.8-fold) and IL-12 (1.7-fold) compared to the infected control. However, combined treatment with INCA-6 and GN 44028 markedly increased TNF-α and IL-12 levels (4.1- and 3.2-fold respectively, compared to the infected control, *p* < 0.0001, F _(4, 35)_ = 86.06 and 41.93, respectively). On the contrary, independent treatment of INCA-6 and GN 44028 moderately reduced IL-10 levels (27.8% and 21.1%, respectively compared to the infected control), while simultaneous treatment markedly reduced the level of IL-10 (42.8% compared to the infected control, *p* < 0.0001, F _(4, 35)_ = 70.25) (Fig. 8*G*). All these observations confirm the importance of the cAMP/EPAC/phospholipase/calcium signaling axis in IL-33 production in VL and further verify the role of IL-33 in modulating cytokine profile to favor the survival of intracellular parasites.

## Discussion

**Figure 9:**
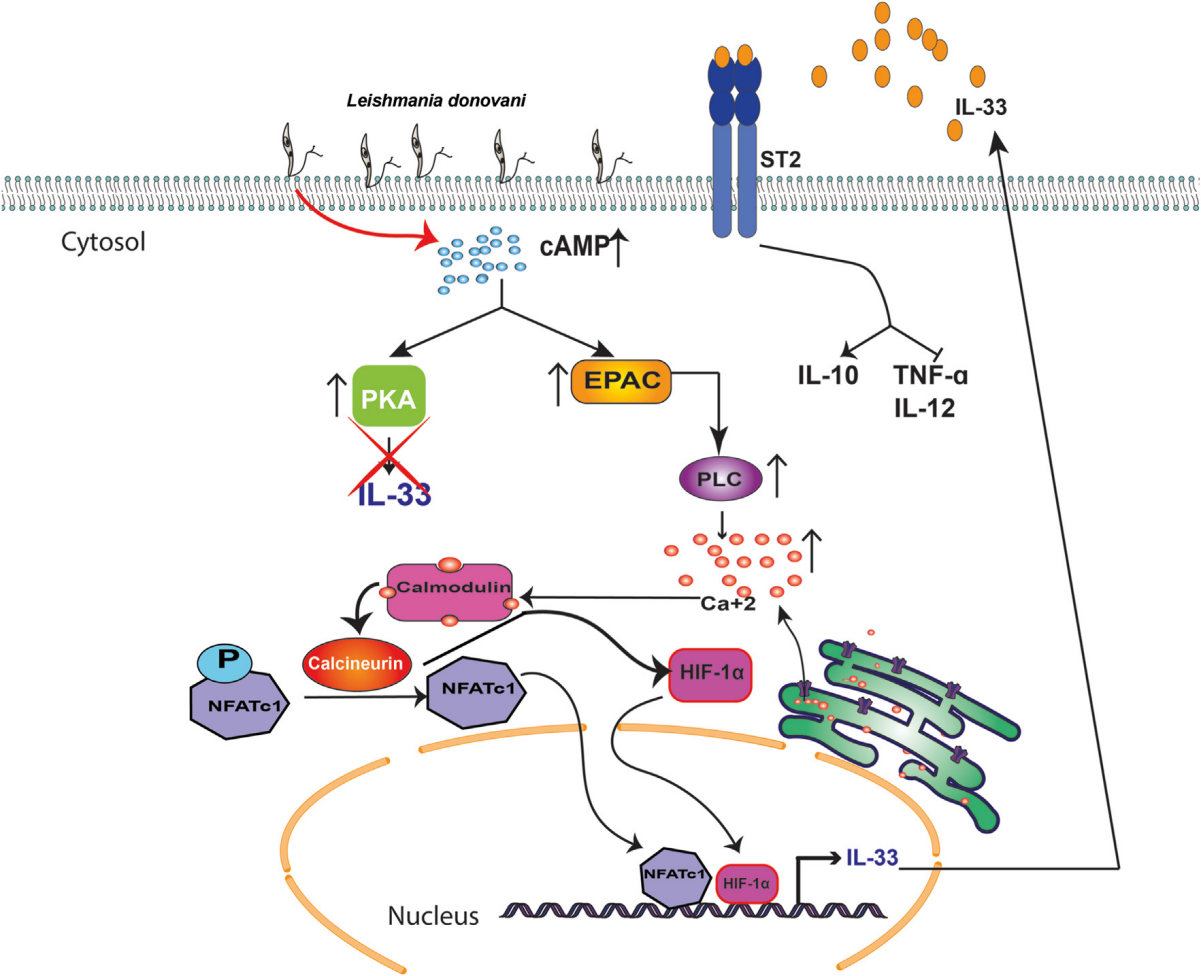
***Leishmania donovani* induces cAMP/EPAC/phospholipase/calcium signaling to generate IL-33 for propagation of infection.***Leishmania* infection leads to upregulation of cAMP production. cAMP then activates EPAC, which further activates PLC. PLC results in elevated intracellular calcium levels, which promotes calcineurin activity by forming a calcium–calmodulin complex. Calcineurin then regulates two downstream transcription factors of IL-33, NFATc1, and HIF-1α, thereby resulting in increased production of IL-33. IL-33 further controls the production of cytokines to create a parasite favorable environment and helps in the propagation of infection. EPAC, exchange protein activated by cAMP; HIF-1α, hypoxia-inducible factor 1 alpha; IL, interleukin; NFATc, nuclear factor of activated T cell; PLC, phospholipase C.

The role of anti-inflammatory cytokines in the establishment and persistence of *L. donovani* infection is a well-trodden area (59, 60, 61, 62). The parasite takes over the host’s immune system and shifts the immune balance toward Th2 responses to make its niche hospitable (63). Knowledge regarding the macrophage signaling pathways modulated by infection provides major advancement toward drug development against the fatal disease. Although IL-10, TGF-β, *etc.* are well-known anti-inflammatory cytokines providing support for the pathogen (64, 65), reports regarding the involvement of IL-33 are scanty. So far only one group has reported the role played by IL-33 in the propagation of visceral infection (17)*.* However, a few reports are there to show that IL-33 can activate both proinflammatory and anti-inflammatory responses under different circumstances (66, 67). The anti-inflammatory function of IL-33 in infection is primarily centered on reducing proinflammatory cytokines. In experimental cerebral malaria, it was found to suppress the production of IL-1β (19) and in *Mycobacterium* infection*;* it exerts its anti-inflammatory activity by reducing the expression of both the IL-1β and inducible nitric oxide synthase (20). Still, the crucial question remains to be answered as to how IL-33 itself is induced and we aimed to address the detailed signaling pathway of IL-33 activation in *Leishmania*-infected macrophages. We found that in infected macrophages, elevated levels of cAMP/EPAC/PLC signaling-mediated calcium release activated two transcription factors NFATc1 and HIF-1α, which are responsible for activating IL-33 transcription (Fig. 9). IL-33 further modulates overall cytokine balance toward anti-inflammatory and creates parasite favorable environment. However, TGF-β, an important anti-inflammatory cytokine, is not regulated by IL-33 during *Leishmania* infection. IL-33–mediated reduction of proinflammatory cytokines could a consequence of increased IL-10 production, which is known to negatively regulate proinflammatory cytokines (64). Increased production of TNF-α and IL-12 during infection in the presence of IL-10 neutralizing antibodies further supports the hypothesis and paves avenue to explore the interdependency of IL-33 and IL-10.

Our earlier study has shown that intracellular cAMP level during *Leishmania* infection plays a major role in deciding the outcome of infection (28). Since a recent report has mentioned the involvement of cAMP in IL-33 production (29), we focused our search on IL-33 signaling starting from the elevated levels of intracellular cAMP in infection. NF-κB and MAPK signaling has previously been demonstrated to produce IL-33 (68, 69), but we chose to ignore them as these signaling pathways are downregulated during active VL (34, 70). cAMP level indeed corroborated with IL-33 production in infection and EPAC and PKA, both the principal downstream effector molecules of cAMP, can facilitate the production of IL-33 in different circumstances (29, 71). Moreover, both these molecules are known for their parasite-favorable role in VL (28). Although PKA gets activated in infection its activity is not stable throughout the infection. CREB, the downstream effector of PKA, is also known to be activated during infection (72). EPAC is well established for activating Rap1 by serving as a GEF (73, 74) and our observation further indicates a link between EPAC-mediated Rap1 activation and the production of IL-33. EPAC has been recognized to regulate other infections such as *Plasmodium* sp., but none was associated with IL-33. Moreover, the cAMP-mediated EPAC pathway gets activated in *Plasmodium falciparum* merozoites rather than within the host cell (75). Despite the activation of PKA during infection, its indifference to IL-33 remains to be a mystery and may be attributed to its irregular activation kinetics. Downstream of EPAC, we found PLC which is known to be a major member of EPAC signaling (40, 41). PLC has previously been reported to be activated in *Leishmania* infection (45), but the identification of the specific isoform involved remains elusive. In addition to its role in the propagation of infection, PLC also serves as a virulence factor in the context of *Trypanosoma brucei* infection (76) and inducer of intracellular calcium level (43, 44). Calcium signaling can activate various downstream pathways such as MAPK cascade (77) and PKC (78). Previously, *L. donovani* infection was also reported to induce intracellular calcium release (45). Although calcium induction propagates *Leishmania* infection, homeostasis needs to be maintained as excess calcium can lead to parasitic death (79). Calmodulin is a calcium sensor with high-affinity Ca2+ binding domains, which acts as a leading component of calcium signaling (47). Calcium/calmodulin complex can activate several downstream kinases and phosphatases (80) out of which calcineurin has been identified as the upstream regulator of IL-33 in our present study. Calcineurin is a calcium and calmodulin-dependent serine/threonine protein phosphatase, which participates in several calcium-dependent signal transduction pathways (81). In addition to its phosphatase activity, calcineurin also serves as an anchoring protein for *P. falciparum* parasites, which utilize it to attach to their host cell (82). To date, several transcription factors associated with IL-33 production have been explored. CREB is involved in the transcriptional regulation of IL-33 (71) and *L. donovani* infection also leads to increased expression of CREB (72). But CREB is generally regulated by PKA (57), which does not regulate IL-33 signaling in VL according to our observation. As mentioned earlier, NF-κB and IRF3 are two TLR-associated transcription factors involved in IL-33 induction (68, 71). However, activation of the TLR pathway is detrimental for the parasite as it facilitates the production of several proinflammatory cytokines such as TNF-α and IL-12 (34). Expression of IL-33 in mast cells is ruled by calcium-dependent transcription factor NFATc1 (55). During the late phase of *L. donovani* infection, expression of PD-1, an NFATc1-targeted gene, gets upregulated indicating its nuclear translocation (83). Another study reported HIF-1α associated production of IL-33 (84). HIF-1α is also a calcium-sensitive transcription factor as its stability is dependent on calcineurin (56). Moreover, the role of HIF-1α was also indicated in favor of infection as it is associated with the induction of the M2 phenotype of myeloid cells (85). Administration of inhibitors of NFATc1 and HIF-1α in infected mice depicted similar observations and justifies the role of these two transcription factors in IL-33 secretion and propagation of infection.

In summary, the present work enlightens how the parasite exploits the host signaling cascade to produce IL-33 for its survival. EPAC, a cAMP-dependent GEF, is a major part of this signaling network, which produces IL-33 in a calcium-dependent manner. In addition to *in vitro* experiments, the role of IL-33 was also verified in the case of *in vivo* study by neutralizing IL-33 in infected BALB/c mice. Treatment with anti-IL-33 antibody resulted in reduced liver and spleen parasite burden, which is also associated with a host-favorable proinflammatory environment. This study, therefore, illustrates the parasite-favorable role of IL-33 in *L. donovani* infection and thus led to the identification of IL-33 as a potential therapeutic target in VL.

## Experimental procedures

### Cells, parasites, and treatments

*L. donovani* strain AG83 (MHOM/IN/1983/AG83), isolated from an Indian patient with Kala-azar (86), was maintained in inbred BALB/c mice by i.v. passage every 6 weeks. *L. donovani* promastigotes were obtained by allowing isolated splenic amastigotes to transform in a parasite growth medium for 72 h at 22 °C. The growth medium consisted of medium 199 (Invitrogen), supplemented with 10% heat-inactivated fetal bovine serum (Invitrogen). Soluble Leishmanial antigen (SLA) was prepared from promastigotes as described earlier (70). It was used at a concentration of 20 μg/ml. The murine macrophage cell line RAW 264.7 (National Repository for Cell Lines/Hybridomas, Department of Biotechnology, Government of India) was maintained at 37 °C, 5% CO_2_ in Dulbecco's Modified Eagle Medium (Invitrogen) supplemented with 10% fetal bovine serum, penicillin (100 U/ml), and streptomycin (100 μg/ml) (Invitrogen) and were regularly monitored for *Mycoplasma* infection. BMDMs were isolated from the femurs and tibiae of euthanized BALB/c mice (6–8 weeks old) (70). *In vitro* infection of macrophages was carried out with *L. donovani* promastigotes at a parasite/cell ratio of 10:1 (34) for specific periods of incubation.

### Reagents

All the inhibitors used in the experiments were obtained from Sigma and Tocris (Table S1). List of antibodies are listed in Table S2.

### Cytokine analysis by ELISA

Levels of different cytokines were measured in cell supernatants after required treatments. Cell culture supernatants were collected and centrifuged at 10,000 rpm for 10 min. Cytokine concentrations were determined using a sandwich ELISA kit (Quantikine M; R&D Systems) and an ELISA plate reader (Bio-Rad) according to the manufacturer’s instructions. Before *in vivo* analysis, spleen cells were stimulated with 20 μg/ml SLA for 48 h. The detection limit of these assays was >6.8, >5.1, >2.5, >4, and >4.6 pg/ml for IL-33, TNF-α, IL12p70, IL-10, and TGF-β, respectively.

### Analysis of mRNA level by semiquantitative PCR

RAW 264.7 and BMDM were subjected to infection and total RNA was isolated from the cells using the RNeasy Mini Kit (Qiagen). The concentration of isolated RNA was measured using a NanoDrop 100 spectrophotometer. RNA was used as a template for complementary DNA synthesis through RT-PCR. The amplification of complementary DNA was performed in a thermal cycler for 40 cycles. Oligonucleotides used for RT-PCR were as follows: for β-actin, 5′-TTGTGATGGACTCCGGAGAC-3 (F)′ and 5′-TGATGTCACGCACGATTTCC-3′(R); for IL-33, 5′-GATGGGAAGAAGCTGATGGTG −3′ (F) and 5′-TTGTGAAGGACGAAGAAGGC-3′ (R); for PLCβ, 5′-CTGAGCGGAGAAGAAAATGG-3′(F) and 5′-ACACAGCGACATCCAGACAG-3′ (R); for PLCγ, 5′-AGATCCGTGAAGTTGCCCAG-3′ (F) and 5′-TCAGCCTTGGTTTCCGGAAA-3′ (R); and for PLCε, 5′-GGAGCCAACGTCTGTCTGAA-3′ (F) and 5′-GAGTTTGGGAGCTGTGTGGA-3′ (R). PCR products were further separated through agarose gel electrophoresis on 1% agarose gels.

### Assessment of intracellular infection

For *in vitro* experiments, cells were plated in tissue culture plates containing coverslips. Cells were then infected with *L. donovani* promastigotes and incubated for indicated periods. After incubation, cells were fixed with methanol and stained with PI (1 μg/ml; Sigma) in PBS along with 10 μg/ml RNaseA (28). At the end of the assay, the number of parasites was determined by observing under a confocal microscope (Carl Zeiss) using a 63× oil immersion objective. Images obtained were analyzed by Image J software (https://imagej.net/ij/).

### cAMP assay

The intracellular cAMP level was measured by using a cAMP assay kit from Sigma-Aldrich according to the manufacturer’s protocol.

### Rap1 activation assay

Rap1 GTP level was measured using Rap1 activation assay kit from Sigma-Aldrich according to the manufacturer’s protocol.

### Calcineurin activity assay

Macrophages were infected for mentioned time periods either in the presence of absence of specific inhibitors. The cells were then harvested, and calcineurin activity was measured using a calcineurin cellular activity assay kit (Abcam) following the manufacturer's instructions. Phosphatase activity was quantified by the detection of free phosphate released from the reaction by measuring the absorbance at 620 nm. In each experimental condition, okadaic acid (OA) and EGTA are utilized and absorbance (*A*) value in presence of (OA) - in presence of (OA + EGTA) documents the calcium-dependent CaN activity.

### Cytotoxicity assay

MTT assay was performed to monitor the effect of various chemical compounds on cell viability. A total of 1 × 10^4^ cells were grown in a 96-well plate and incubated overnight. The cells were treated with respective compounds and incubated. MTT (5 mg/ml) was then added and incubated at 37 °C for 4 h. Thereafter, formazan crystals were solubilized in solubilization buffer, and absorbance was measured at 570 nm. The extent of cell viability was measured as the percentage of viability in comparison with the untreated cells.

### Immunoblotting

After indicated treatments and infections, cells were lysed using ice-cold lysis buffer (Cell Signaling Technology), and the protein concentrations in the cell lysates were estimated using the Bradford assay (87). Proteins (50 μg) were then resolved by 10% SDS-PAGE and then transferred to a nitrocellulose membrane (Millipore). Five percent bovine serum albumin in Tris-buffered saline solution was used for blocking the membrane and followed by incubation with primary antibody overnight at dilution recommended by the manufacturer. After washing with wash buffer (Tris-buffered saline-T), membranes were probed with horseradish peroxidase–conjugated secondary antibody for 1 h and detected by chemiluminescence using ECL solution (Bio-Rad). Quantification of band intensities was conducted using the ImageJ software. β-Actin was used as a loading control.

### Immunoprecipitation

Immunoprecipitation was performed as described earlier (34). Briefly, cell lysates were prepared and a small amount of cell lysate was kept separately before antibody addition to find out initial amounts of the test protein. The remaining lysate was incubated overnight with a specific primary antibody at 4 °C. 25 μl of protein A/G plus agarose beads (Santa Cruz Biotechnology) were then added to the solution and incubated for 4 h at 4 °C. Immune complexes were separated and washed three times with ice-cold lysis buffer and once with lysis buffer without Triton X-100. The immunoprecipitated samples and cell lysates were resolved through 10% SDS-PAGE. The proteins were then immunoblotted as previously mentioned.

### RNA-mediated interference by siRNA transfection

Transfection was carried out with control/specific siRNAs (Table S3). The sequences were kindly designed by Dr Ajit Chande and Pratibha Madbhagat, IISER Bhopal. Following transfection, knockdown efficiency was verified by Western blotting. siRNA with comparatively higher knockdown efficiency was utilized for the study.

### PLC activity assay

PLC activity was measured using a PLC activity assay kit from Abcam, according to the manufacturer’s instructions.

### ChIP assay

Cells were cross-linked with 1% formaldehyde, harvested in lysis buffer (1% SDS, 10 mM EDTA, 50 mM Tris–HCl, pH 8.0, and 1× protease inhibitor mixture), and sonicated, followed by immunoprecipitation with antibodies. Immunoprecipitation with a normal rabbit IgG served as a negative control. Immunoprecipitated cell lysates were incubated with protein A/G plus agarose, washed, and then heated at 65 °C for 1.5 h to reverse the cross-linking. DNA fragments were purified, and PCR amplification was performed using 5 μl of DNA (recovered from ChIP) with 35 cycles of denaturation, annealing, and extension and amplified PCR products were analyzed by electrophoresis on a 1% agarose gel. The following primer pairs were used to amplify putative IL-33 promoter regions, 5′-CCTTCACTACCACTCACCCC-3' (sense) and 5′-GATCGGGGCCAACTTTTCTC-3′ (antisense).

### Isolation of nuclear fraction

To prepare subcellular fractions, the cells were lysed through a 10-min hypotonic treatment on ice in buffer A (10 mM Hepes [pH 7.9], 10 mM KCl, 1.5 mM MgCl_2_, 0.5 mM DTT, 0.5 mM PMSF, 10 μg of leupeptin per ml, 10 μg of pepstatin per ml, and 0.01 U of aprotinin per ml), followed by homogenization using a narrow-gauge syringe. The solution was then centrifuged at 4 °C for 10 min at 10,000*g* and the supernatant was collected as the cytosolic extract. The pellet was washed once with ice-cold buffer A and resuspended in two volumes of buffer B (20 mM Hepes [pH 7.9], 0.42 M NaCl, 1.5 mM MgCl_2_, 0.2 mM EDTA, 0.5 mM DTT, 0.5 mM PMSF, 10 μg of leupeptin per ml, 10 μg of pepstatin per ml, 0.01 U of aprotinin per ml, and 25% glycerol). After the concentration of NaCl was adjusted to 0.38 M, the suspension was placed at −70 °C for 10 min, thawed slowly on ice, and then incubated for 10 min in ice with intermittent tapping. After a 15-min centrifugation at 10,000*g* and 4 °C, the supernatant solution representing the nuclear fraction was isolated.

### Fluorescence microscopy

Macrophages were plated onto coverslips and cultured overnight. The cells were treated as mentioned and infected with *L. donovani* promastigotes, washed twice in PBS, and fixed with 4% paraformaldehyde for 30 min at room temperature. The cells were permeabilized with 0.1% Triton X-100 and incubated with blocking solution, followed by primary antibody overnight at 4 °C. After washing, coverslips were incubated with fluorescent dye–conjugated secondary antibodies for 1 h at room temperature. The cells were stained with 4′,6-diamidino-2-phenylindole (1 μ*g*/ml) in PBS plus 10 μ*g*/ml RNase A to label the nucleus, mounted on slides, and visualized under confocal microscope (Carl Zeiss) using 63× oil immersion objective. Images obtained were analyzed by Image J software.

### *In vivo* infection

Animal maintenance and experiments were performed following the guidelines provided by the Committee for the Purpose of Control and Supervision of Experiments on Animals. The protocol was approved by the Departmental Animal Ethics Committee (Institutional Animal Ethics Committee, Department of Biochemistry, University of Calcutta). For *in vivo* infection, 6- or 8-week-old female BALB/c mice (∼20 g) were maintained in temperature controlled environment with a 12 h light/12 h dark cycle and provided with a standard diet and water *ad libitum.* Mice were kept in a pathogen-free room at the animal house of the institute for more than 1 week before experimental infection. All procedures were performed according to the protocol approved by the Institutional Animal Ethics Committee. Mice were injected *via* the tail vein with 1 × 10^7^ stationary phase *L. donovani* promastigotes as described earlier (34). At the 10th day postinfection, mice were intraperitoneally administered with either INCA 6 or GN 44028 or both at a dose of 10 mg/kg body weight/day at every fifth day till 45 days postinfection. Infection was assessed by removing the liver and spleen from infected mice. Liver and spleen weights were monitored using an electronic precision balance. Parasite burden was determined from Giemsa-stained impression smears (88). Liver and spleen parasite burdens, expressed as Leishman-donovan units, were calculated as the number of amastigotes/1000 nucleated cells × organ weight (in grams) (89). Throughout the experimental time, animals were checked for body weight, activity, and body temperature. Splenocytes from BALB/c mice were isolated and cultured as described previously. Briefly, spleens were aseptically removed from mice and teased into single-cell suspensions in Roswell Park Memorial Institute 1640 supplemented with penicillin (100 U/ml), streptomycin (100 μg/ml), 2-mercaptoethanol (50 μM), L-glutamine (2 μM), Hepes (10 μM), and 10% Fetal bovine serum. Red blood cells were separated by lysis with 0.83% NH_4_Cl. The remaining cells were washed twice with a culture medium. Splenocyte suspensions (1 × 10^6^ cells/ml) were taken into 35-mm tissue culture plates and incubated at 37 °C in 5% CO2. Adherent cells were then stimulated with SLA, and levels of various cytokines were measured by ELISA (90).

### Histopathology

Isolated livers were fixed in 10% formalin (Merck) and embedded in paraffin wax. Tissue sections (5 mm) were made with microtome (Leica Biosystems) and stained with H&E to study their microarchitecture by light microscopy (91).

### Densitometric analysis

Densitometric analysis for all the experiments was carried out using ImageJ software. Band intensities were quantitated densitometrically, and the values obtained were normalized to endogenous control and expressed in arbitrary densitometric units as indicated in bar graphs adjacent to the figures.

### Statistical analysis

Experiments were performed for indicated times as mentioned in the Figure legend. Data are shown as mean ± SD from the indicated number of independent experiments. Statistical analysis was carried out using GraphPad Prism 8.0.1 Software. For comparison between two groups, Student’s *t* test was used, and for comparison between three or more groups, one-way or two-way ANOVA with Tukey post hoc test was used. To assess the statistical differences among pairs of datasets, a *p* value of <0.05 is considered to be significant. Western blot quantitation was performed using ImageJ software.

## Data availability

All data are included in the manuscript.

## Supporting information

This article contains supporting information.

## Conflict of interest

The authors declare that they have no conflicts of interest with the contents of this article.
