# Supplementary material for: Leishmania exploits host cAMP/EPAC/calcineurin signaling to induce an IL-33–mediated anti-inflammatory environment for the establishment of infection
Source: J Biol Chem. 2024 May 13;300(6):107366. doi: 10.1016/j.jbc.2024.107366 (PMC11208913; doi:10.1016/j.jbc.2024.107366)
Supplement: Supporting Figures S1–S5 and Tables S1–S3 [file mmc1.docx]

## *Leishmania*exploits host cAMP/EPAC/Calcineurin signaling to induce an IL-33-mediated anti-inflammatory environment for the establishment of infection

## Souravi Roy^1^, Shalini Roy^1^, Satyajit Halder^2^, Kuladip Jana^2^ and Anindita Ukil^1*^

## ^1^Department of Biochemistry, University of Calcutta, Kolkata, India.

^2^Division of Molecular Medicine, Bose Institute, Kolkata, India

**Running Title:** EPAC/calcineurin signaling regulates IL-33 in infection

^*^Address correspondence:

Dr. Anindita Ukil

Department of Biochemistry

Calcutta University

35, Ballygunge Circular Road

Kolkata 700019, India.

Phone: 91-33-2461-5455Fax: 91-33-2461-4849

E-mail address[: u.anindita@gmail.com](mailto::%20u.anindita@gmail.com)

**Supplementary Figures**

**
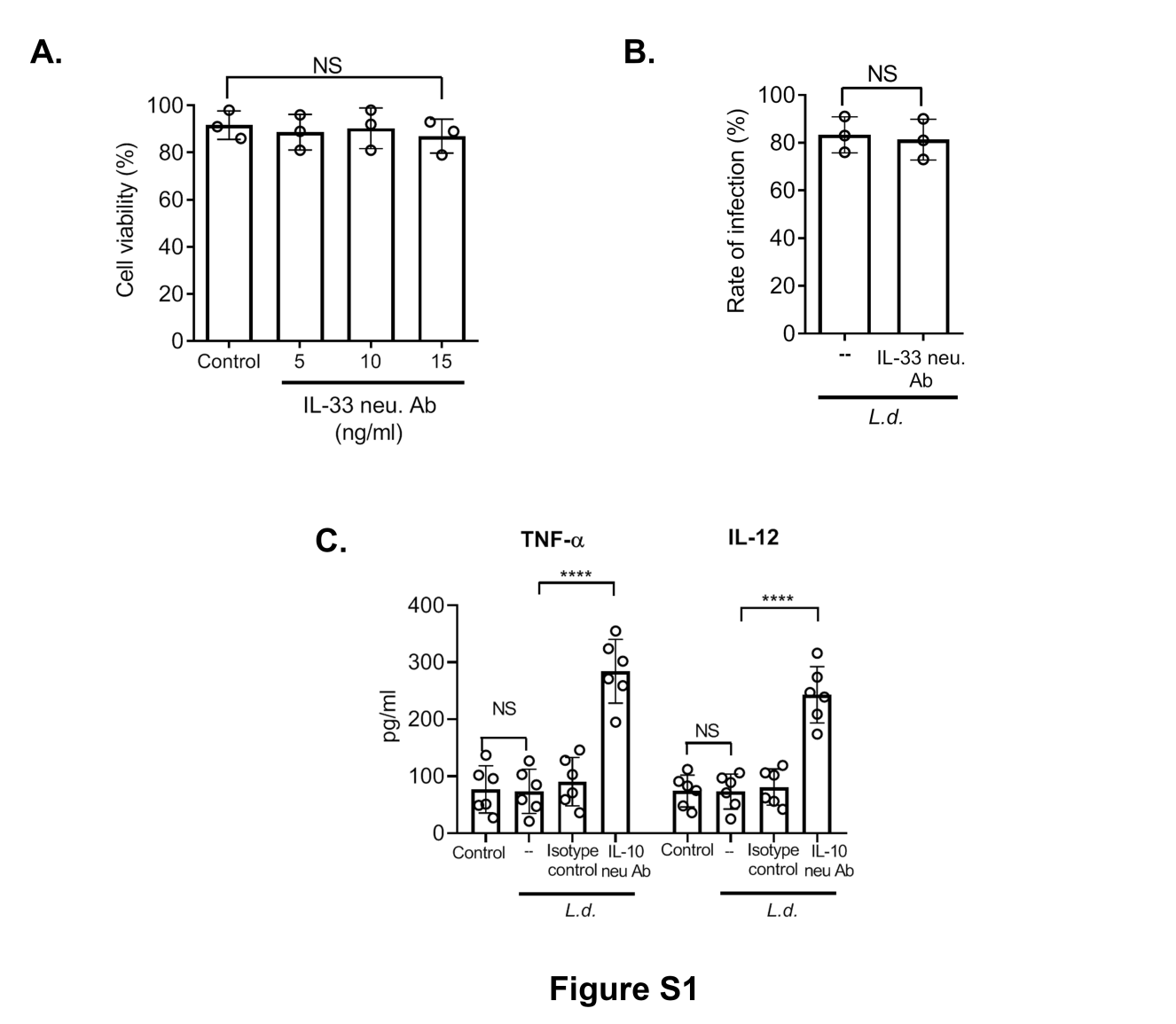
**

**Figure S1.** IL-33 neutralizing antibody exerts no effect on host cell viability or rate of infection. (A) RAW 264.7 cells were incubated with increasing concentrations of IL-33 neutralizing antibody and cell viability was determined by MTT assay (n=3). (B) RAW 264.7 macrophages were infected with *L. donovani* promastigotes for 4 h either in the presence or absence of IL-33 neutralizing antibody. The number of parasites per 100 macrophages was determined by PI staining (n=3). (C) Culture supernatants of macrophages infected with *L. donovani* promastigotes in the presence or absence of IL-10 neutralizing antibody for 48 h were assayed for TNF-α and IL-12 by ELISA (n=6). The graph shows the combined (mean) outcomes from indicated number of independent experiments, and the error bars indicate the variation between those independent repeats (mean ± SD); NS, not significant, **P*<0.05, ***P*<0.01, ****P*<0.001, *****P*<0.0001 (Student’s t test and ANOVA with Tukey *post hoc* test).

**
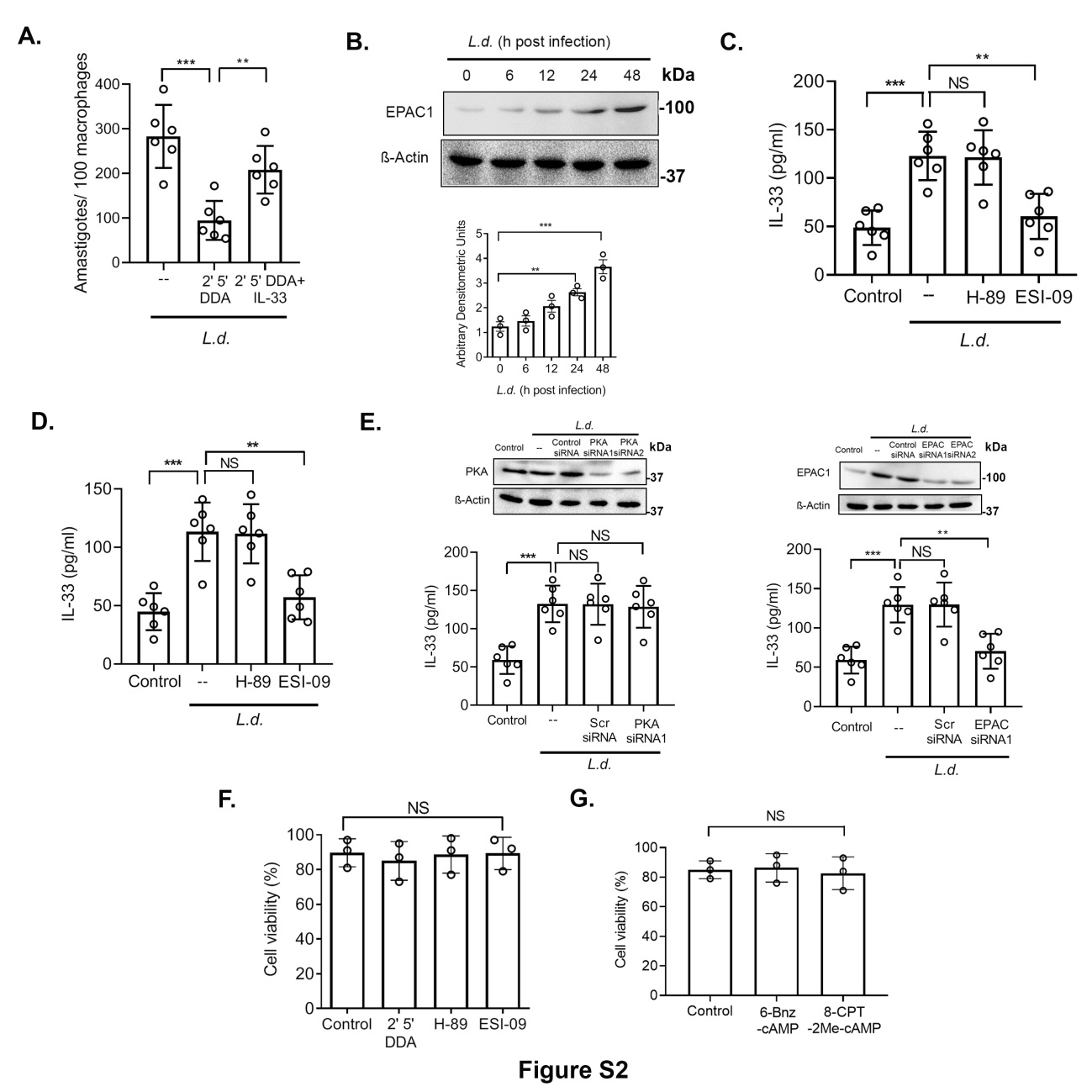
**

**Figure S2.** cAMP-EPAC pathway is responsible for infection-induced IL-33 production.

(A) *L. donovani-*infected macrophages were treated with cAMP inhibitor 2’ 5’ DDA either in the presence or absence of recombinant IL-33 and intracellular parasite numbers were determined (n=6). (B) BMDM were infected with *L. donovani* promastigotes for the indicated time periods. The expression of EPAC1 was evaluated at the protein level by immunoblotting (n=3). (C) Macrophages were infected with *L. donovani* promastigotes for 48 h, pre-treated either with H-89 (10 µM) or ESI-09 (10 µM), and IL-33 level was determined by ELISA (n=6). (D) BMDM were infected with *L. donovani* promastigotes in the presence of either H-89 (10 µM) or ESI-09 (10 µM) and IL-33 level was measured by ELISA (n=6). (E) Macrophages were transfected with either PKA siRNA or EPAC siRNA and then infected with *L. donovani* promastigotes for 48 h and the culture supernatants were assayed for IL-33 by ELISA. (n=6) (F) Macrophages were treated with 2’ 5’ DDA (100 µM), H-89 (10 µM), and ESI-09 (10 µM) independently and cell viability was monitored (n=3). (G) Macrophages were treated with 6-Bnz-cAMP (50 µM) and 8-CPT-2Me-cAMP (50 µM) independently and cell viability was monitored (n=3). The graph shows the combined (mean) outcomes from indicated number of independent experiments, and the error bars indicate the variation between those independent repeats (mean ± SD); NS, not significant, **P*<0.05, ***P*<0.01, ****P*<0.001, *****P*<0.0001 (ANOVA with Tukey *post hoc* test).

**
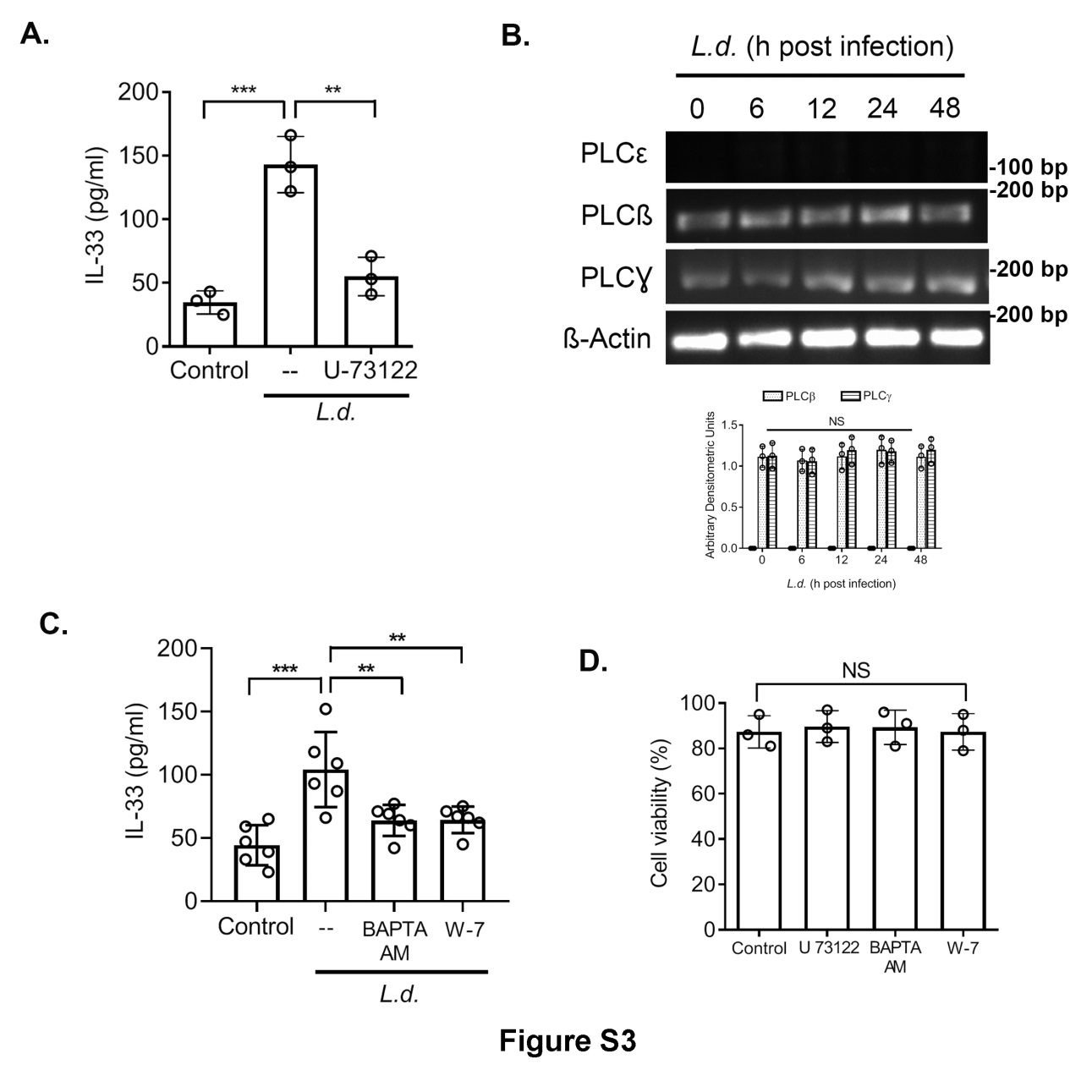
Figure S3.** EPAC-induced intracellular calcium leads to IL-33 production during infection.

(A) Infected BMDM were pre-treated with U 73122 (10 µM) and IL-33 levels were measured (n=3). (B) mRNA levels of different PLC isoforms PLCε, PLCβ and PLCγ were assessed by RT-PCR in infected RAW 264.7 cells (n=3). (C) IL-33 level was analyzed in infected BMDM either in the absence or presence of BAPTA AM (10 µM) or W-7 hydrochloride (10 µM) (n=6). (D) RAW 264.7 cells were treated with U 73122 (10 µM), BAPTA AM (10 µM), or W-7 hydrochloride (10 µM) independently and cell viability was monitored (n=3). The graph shows the combined (mean) outcomes from indicated number of independent
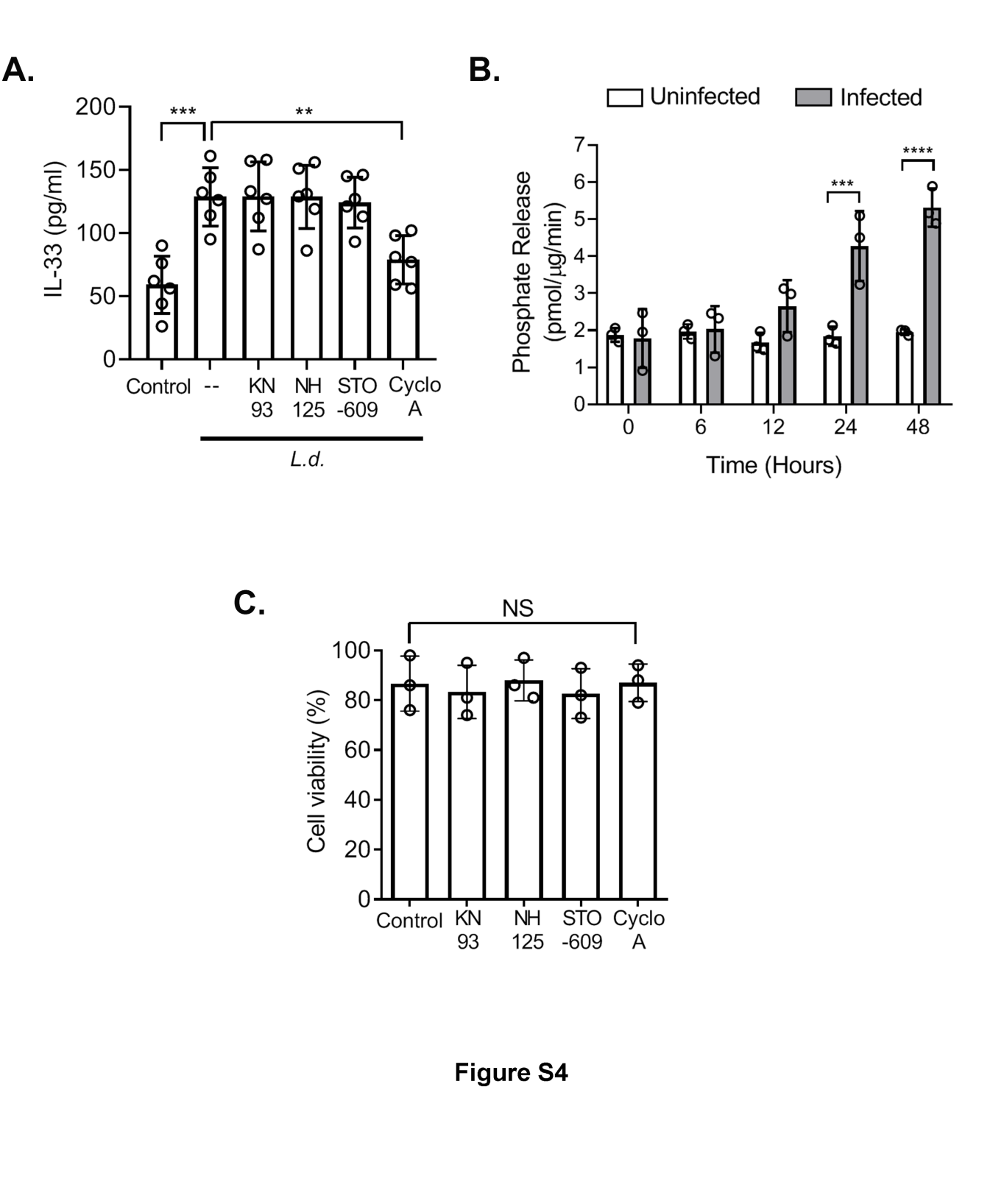
experiments, and the error bars indicate the variation between those independent repeats (mean ± SD); NS, not significant, **P*<0.05, ***P*<0.01, ****P*<0.001, *****P*<0.0001 (ANOVA with Tukey *post hoc* test).

**Figure S4.** Calcium-dependent calcineurin activation is required for IL-33 production post-infection. (A) BMDM were incubated with KN93 (1 µM), NH 125 (3 µM), STO-609 acetate (10 µM), or cyclosporin A (1 µg/ml) prior to infection with *L. donovani* promastigotes, and IL-33 levels were determined (n=6). (B) The activity of calcineurin was measured in BMDM post-*L. donovani* infection (0-48 h) (n=3). (C) RAW 264.7 cells were incubated with mentioned doses of inhibitors and cell viability was determined by MTT assay (n=3). The graph shows the combined (mean) outcomes from indicated number of independent experiments, and the error bars indicate the variation between those independent repeats (mean ± SD); NS, not significant, **P*<0.05, ***P*<0.01, ****P*<0.001, *****P*<0.0001 (ANOVA with Tukey *post hoc* test).

**
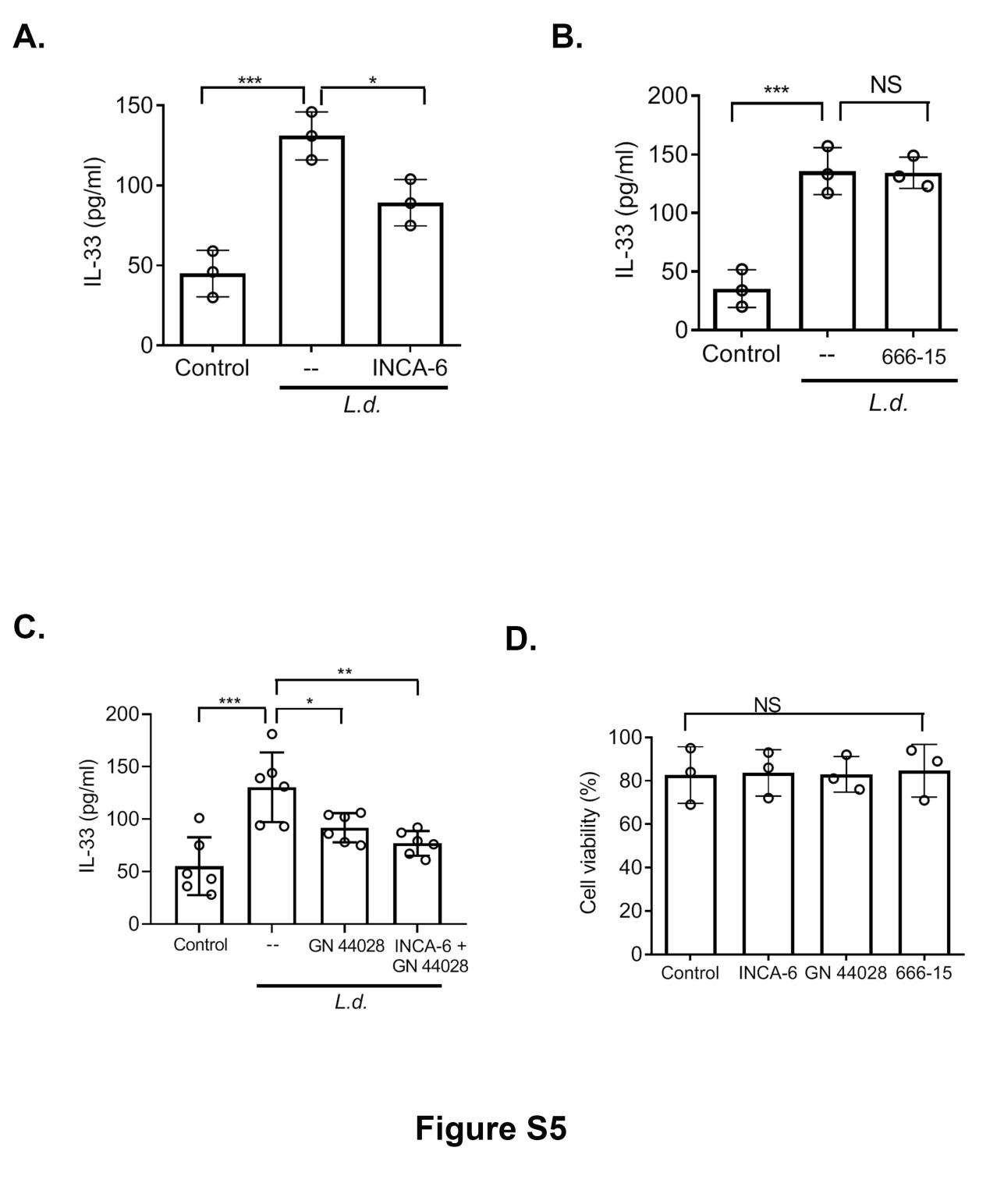
**

**Figure S5.** Calcineurin-dependent transcription factors NFATc1 and HIF-1α are involved in IL-33 production during infection. (A) BMDM were treated with INCA-6 prior to infection and IL-33 secretion was measured by ELISA (n=3). (B) RAW 264.7 cells were infected with *L. donovani* promastigotes either in the presence or absence of CREB inhibitor 666-15 and IL-33 level was measured (n=3). (C) BMDM were pre-treated either with GN44028 (10 µM) alone or in combination with INCA-6 (10 µM) followed by infection with *L. donovani* and the level of IL-33 was monitored (n=6). (D) RAW 264.7 cells were treated with INCA-6 (10 µM), GN44028 (10 µM) or 666-15 (5µM) and cell viability was determined by MTT assay (n=3). The graph shows the combined (mean) outcomes from indicated number of independent experiments, and the error bars indicate the variation between those independent repeats (mean ± SD); NS, not significant, **P*<0.05, ***P*<0.01, ****P*<0.001, *****P*<0.0001 (ANOVA with Tukey *post hoc* test).

**Table 1. List of inhibitors used in experiments.**

| **Inhibitors** | **Source** | **Catalogue No.** | **Concentration** |
| --- | --- | --- | --- |
| 2’ ,5’-Dideoxyadenosine (adenylate cyclase inhibitor) | Sigma-Aldrich | D7408 | 100 µM |
| H-89 dihydrochloride  (Protein Kinase A inhibitor) | Sigma-Aldrich | B1427 | 10 µM |
| ESI-09 (EPAC inhibitor) | Sigma-Aldrich | 5.00506.0001 | 10 µM |
| U 73122 (Phospholipase C inhibitor) | Tocris | 1268 | 10 µM |
| BAPTA, AM (cell permeant calcium chelator) | Tocris | 2787 | 10 µM |
| W-7 hydrochloride  (Calmodulin inhibitor) | Tocris | 0369 | 10 µM |
| KN 93 (CaM kinase II inhibitor) | Tocris | 5215 | 1 µM |
| NH 125 (CaM kinase III inhibitor) | Tocris | 3439 | 3 µM |
| STO-609 acetate (CaM kinase kinase inhibitor) | Tocris | 1551 | 10 µM |
| Cyclosporin A (Calcineurin inhibitor) | Sigma-Aldrich | SML1018 | 1 µg/ml |
| INCA-6 (Inhibitor of calcineurin-substrate association) | Tocris | 2162 | 10 µM |
| GN 44028 (HIF-1α inhibitor) | Tocris | 5655 | 10 µM |
| 666-15 (CREB inhibitor) | Tocris | 5661 | 5 µM |

**Table 2. List of antibodies used in experiments.**

| **Antibody** | **Source** | **Catalogue no.** | **Dilution** |
| --- | --- | --- | --- |
| EPAC1 (5D3) Mouse mAb | Cell signaling technology | 4155 | 1:1000 |
| PKA C-α Antibody | Cell signaling technology | 4782 | 1:1000 |
| Phospho-CREB (Ser133) (87G3) Rabbit mAb | Cell signaling technology | 9198 | 1:1000 |
| CREB (86B10) Mouse mAb | Cell signaling technology | 9104 | 1:1000 |
| Calcineurin A Polyclonal Antibody | Invitrogen | PA5-17446 | For WB 1:1000  For IP 1:50 |
| Calmodulin Monoclonal Antibody | Invitrogen | MA3-917 | 1:500 |
| NFATC1 Monoclonal Antibody | Invitrogen | MA3-024 | For WB 1:2000  For IF 1:100 |
| HIF1A Polyclonal Antibody | Invitrogen | PA1-16601 | For WB 1:1000  For IF 1:100 |
| Goat Anti-Rabbit IgG H&L (Texas Red ) | Abcam | ab6719 | 1:1000 |
| Goat Anti-Mouse IgG H&L (Texas Red) | Abcam | ab6787 | 1:1000 |
| Anti-Lamin A antibody | Abcam | ab8980 | 1:1000 |
| Monoclonal Anti-β-Actin antibody produced in mouse | Sigma | A2228 | 1:10000 |
| Anti-Mouse IgG (whole molecule)–Peroxidase antibody produced in goat | Sigma | A4416 | 1:10000 |
| Anti-Rabbit IgG (whole molecule)–Peroxidase antibody produced in goat | Sigma | A0545 | 1:10000 |

**Table 3. Sequence of siRNAs.**

| PKA siRNA | 1. 5’-UUUGAGUUGUCCUUGAAGGAG-3’  2. 5’-UUUAAACUUUGGUAUGAAGGG-3’ |
| --- | --- |
| EPAC siRNA | 1. 5’-AGUUGAUGAGGUUCUCUACCA-3’  2. 5’-AGAACUAAGACCACUUUGCCG-3’ |
